# Unmasking of CgYor1-Dependent Azole Resistance Mediated by Target of Rapamycin (TOR) and Calcineurin Signaling in Candida glabrata

**DOI:** 10.1128/mbio.03545-21

**Published:** 2022-01-18

**Authors:** Sonam Kumari, Mohit Kumar, Brooke D. Esquivel, Mohd Wasi, Ajay Kumar Pandey, Nitesh Kumar Khandelwal, Alok K. Mondal, Theodore C. White, Rajendra Prasad, Naseem A. Gaur

**Affiliations:** a Yeast Biofuel Group, International Centre for Genetic Engineering and Biotechnology, New Delhi, India; b Amity Institute of Biotechnology and Integrative Science and Health, Amity University Gurgaon, Haryana, India; c School of Biological and Chemical Sciences, University of Missouri at Kansas City, Kansas City, Missouri, USA; d School of Life Sciences, Jawaharlal Nehru University, New Delhi, India; University of Toronto

**Keywords:** ABC transporters, azole drug resistance, calcineurin, *Candida glabrata*, TOR pathway

## Abstract

In this study, 18 predicted membrane-localized ABC transporters of Candida glabrata were deleted individually to create a minilibrary of knockouts (KO). The transporter KOs were analyzed for their susceptibility toward antimycotic drugs. Although Cg*YOR1* has previously been reported to be upregulated in various azole-resistant clinical isolates of C. glabrata, deletion of this gene did not change the susceptibility to any of the tested azoles. Additionally, Cg*yor1*Δ showed no change in susceptibility toward oligomycin, which is otherwise a well-known substrate of Yor1 in other yeasts. The role of CgYor1 in azole susceptibility only became evident when the major transporter Cg*CDR1* gene was deleted. However, under nitrogen-depleted conditions, Cg*yor1*Δ demonstrated an azole-susceptible phenotype, independent of CgCdr1. Notably, Cg*yor1Δ* cells also showed increased susceptibility to target of rapamycin (TOR) and calcineurin inhibitors. Moreover, increased phytoceramide levels in Cg*yor1*Δ and the deletions of regulators downstream of TOR and the calcineurin signaling cascade (Cg*ypk1*Δ, Cg*ypk2Δ*, Cg*ckb1*Δ, and Cg*ckb2*Δ) in the Cg*yor1*Δ background and their associated fluconazole (FLC) susceptibility phenotypes confirmed their involvement. Collectively, our findings show that TOR and calcineurin signaling govern CgYor1-mediated azole susceptibility in C. glabrata.

## INTRODUCTION

The most widespread human fungal pathogens belong to the genus *Candida*. Candida glabrata is a commensal fungus in the human microbiota but is also known to cause superficial to serious life-threatening bloodstream infections (1). Azoles are first-line antifungals that are used to prevent fungal growth by interfering with ergosterol biosynthesis (2). Fluconazole (FLC), the most commonly used azole, targets 14-α-sterol-demethylase (Erg11) and prevents lanosterol conversion to ergosterol (3). The efficacy of the azoles in the treatment of invasive candidiasis is severely hampered by azole resistance that frequently develops in C. glabrata (4, 5). To overcome azole resistance in C. glabrata, it is first essential to comprehensively understand the mechanisms by which this species confers resistance to the azoles. Canonical mechanisms of azole drug resistance in C. glabrata include mutations in the azole target genes, gain of function (GOF) mutations in the transcription factor (TF) gene Cg*PDR1*, overexpression of efflux pump-encoding genes, and mutations in DNA repair pathway genes (5, 6). Unlike in C. albicans, substitution mutations in Cg*ERG11* are a less common mechanism of azole resistance in clinical isolates of C. glabrata. However, a few mutations in Cg*ERG11*, such as G944A and I166S, have been reported. These Cg*ERG11* mutations exhibit cross-resistance to both azoles and polyenes (7, 8). The TF CgPdr1 is a central player in the azole resistance acquired during antifungal therapy. Notable reports have shown that C. glabrata CgPdr1 directly binds to FLC, which results in activation of drug efflux pumps (9, 10). Moreover, GOF mutations in the putative functional domains of the TF CgPdr1 result in upregulated transcription of ABC transporter coding genes Cg*CDR1*, Cg*PDH1*, and Cg*SNQ2* and increased resistance to azoles (5, 11, 12).

Independent studies have established that ABC drug transporters are major contributors to azole resistance in clinical isolates of C. glabrata. For instance, resistant clinical isolates of C. glabrata show an increased level of Cg*CDR1* transcript and became azole-susceptible upon Cg*CDR1* deletion. Reintroduction of Cg*CDR1* restored the resistance phenotype (13). The expression of Cg*PDH1*, a close homolog of Ca*CDR2*, was also found to be increased in azole-resistant clinical isolates of C. glabrata (14). Further study showed that the deletion of Cg*PDH1* in the Cg*cdr1Δ* strain could increase susceptibility to FLC (15). Thus, both Cg*CDR1* and Cg*PDH1* in tandem or independently contribute to azole resistance in C. glabrata. A member of the PDR transporter family, CgSnq2 was also shown to contribute to azole resistance (5). A study has shown increased expression of Cg*SNQ2* in two azole-resistant clinical isolates in which Cg*CDR1* or Cg*PDH1* expression remained unchanged (16). In one of these isolates, Cg*SNQ2* was required for the azole-resistant phenotype (17). Similar studies have demonstrated the role of other ABC transporters in azole resistance, e.g., CgAus1, CgYor1, and CgYbt1. CgAus1 protects cells against azole antifungals in the presence of serum (17–19). The transcript levels of Cg*YOR1* and Cg*YBT1* were found to be upregulated in azole-resistant lab mutants as well as in azole-resistant clinical isolates (20). A previous report also suggested the upregulation of Cg*YCF1*, Cg*YBT1*, and Cg*YOR1* in azole-resistant petite isolates (21).

Pathogenic fungi have also evolved a multitude of stress response pathways that enable cells to tolerate azoles. Key regulators of drug-induced stress are target of rapamycin (TOR) kinase, Hsp90, and calcineurin. It has been established that ABC drug transporters impart drug resistance by drug extrusion from the cell, allowing cells to withstand antifungals. Interestingly, a recent study confirmed that hyper-activation of TOR signaling could significantly contribute to azole resistance in the absence of ABC transporter CaCdr6 in C. albicans (22). Noteworthy reports also revealed the elegant role of the TOR cascade, the molecular chaperone Hsp90 and its downstream targets, and calcineurin stabilization in enabling cellular stress responses crucial for the emergence and maintenance of drug resistance in Aspergillus and *Candida* species (23, 24).

We have recently inventoried 25 ABC proteins in C. glabrata to show that 18 ABC proteins are predicted to be membrane-localized and could be drug transporters. The change in transcriptional response to drug exposure highlighted their roles in C. glabrata (25). The role of some of the ABC transporters as major azole transporters has been established; however, the existence of a large number of ABC transporter members in the C. glabrata genome prompted us to explore their involvement in azole resistance. For this, we created a minilibrary of deletions for all 18 ABC transporter members in haploid C. glabrata. Individual transporter deletions had no consequence upon susceptibility toward tested azoles and other drugs except in the cases of Cg*cdr1*Δ, Cg*snq2*Δ, and Cg*atm*1Δ, where their disruptions increased susceptibility to few tested drugs.

Strikingly, our double deletions of ABC transporter-encoding genes unmasked the contribution of CgYor1 in azole resistance of C. glabrata. Notably, the transcript level of Cg*YOR1* was shown to be higher in various resistant clinical isolates (20); however, there was no direct link for this member of the multidrug resistance-associated protein (MRP) family to azole resistance. Our study shows for the first time that oligomycin (OMY), a well-known substrate of Yor1 in Saccharomyces cerevisiae and C. albicans, is not a substrate of CgYor1. Instead, CgYor1 imparts azole resistance. Additionally, we report that the repression of TOR and calcineurin signaling and change in sphingolipid compositions in Cg*yor1*Δ are directly associated with the azole-susceptible phenotype.

## RESULTS

### Single deletions of ABC membrane transporters have no impact on cell growth.

To construct a minilibrary of 18 ABC membrane transporter knockouts in C. glabrata, single gene deletions were created using a fusion PCR-based method employing a dominant selection marker *NAT1* gene as described previously (26, 27). We assessed if removing a transporter from the genome affects the growth kinetics of the resulting mutant. We employed the microtiter plate-based growth assay to record the growth of all constructs (27). The doubling time of 60 min was recorded for most of the mutants belonging to pleotropic drug resistance (PDR), MRP, and adrenoleukodystrophy (ALDp) subfamilies, which were comparable to the wild-type (WT) cells (Fig. S1a and b). However, Cg*atm1*Δ, a member of the multidrug resistance (MDR) subfamily of transporters, displayed slower growth with a doubling time of 80 min and grew as petite colonies. CgAtm1 is predicted to be localized in the mitochondrial membrane and involved in ATP-dependent Fe-S cluster translocation. The deletion of the gene might be linked to perturbation of mitochondrial function, making the cells petite, which was confirmed by the inability of the mutant to grow on yeast extract peptone glycerol (YPG) medium (Fig. S1c).

10.1128/mbio.03545-21.1 FIG S1 Growth curve and doubling time analysis of all deletion strains in YPD. (a) Growth pattern of ABC transporter mutants. (i) PDR subfamily mutants. (ii) MDR subfamily mutants. (iii) MRP subfamily mutants. (iv) ALDp subfamily mutants. (b) Doubling time determination of the mutants of ABC transporters. Except for mutant *Cgatm1*Δ, belonging to the MDR subfamily, which showed a growth defect with a doubling time of 80 min, the rest of the mutants have a doubling time of 60 minutes. (c) Growth of Cg*atm1*Δ on agar media containing glycerol as the sole carbon source. Download FIG S1, PPTX file, 0.4 MB. Copyright © 2022 Kumari et al. 2022 Kumari et al. https://creativecommons.org/licenses/by/4.0/ This content is distributed under the terms of the Creative Commons Attribution 4.0 International license.

### Except for Cg*cdr1*Δ, Cg*snq2Δ*, and Cg*atm1Δ*, none of the ABC transporter mutants exhibit susceptibility to tested drugs.

**FIG 1:**
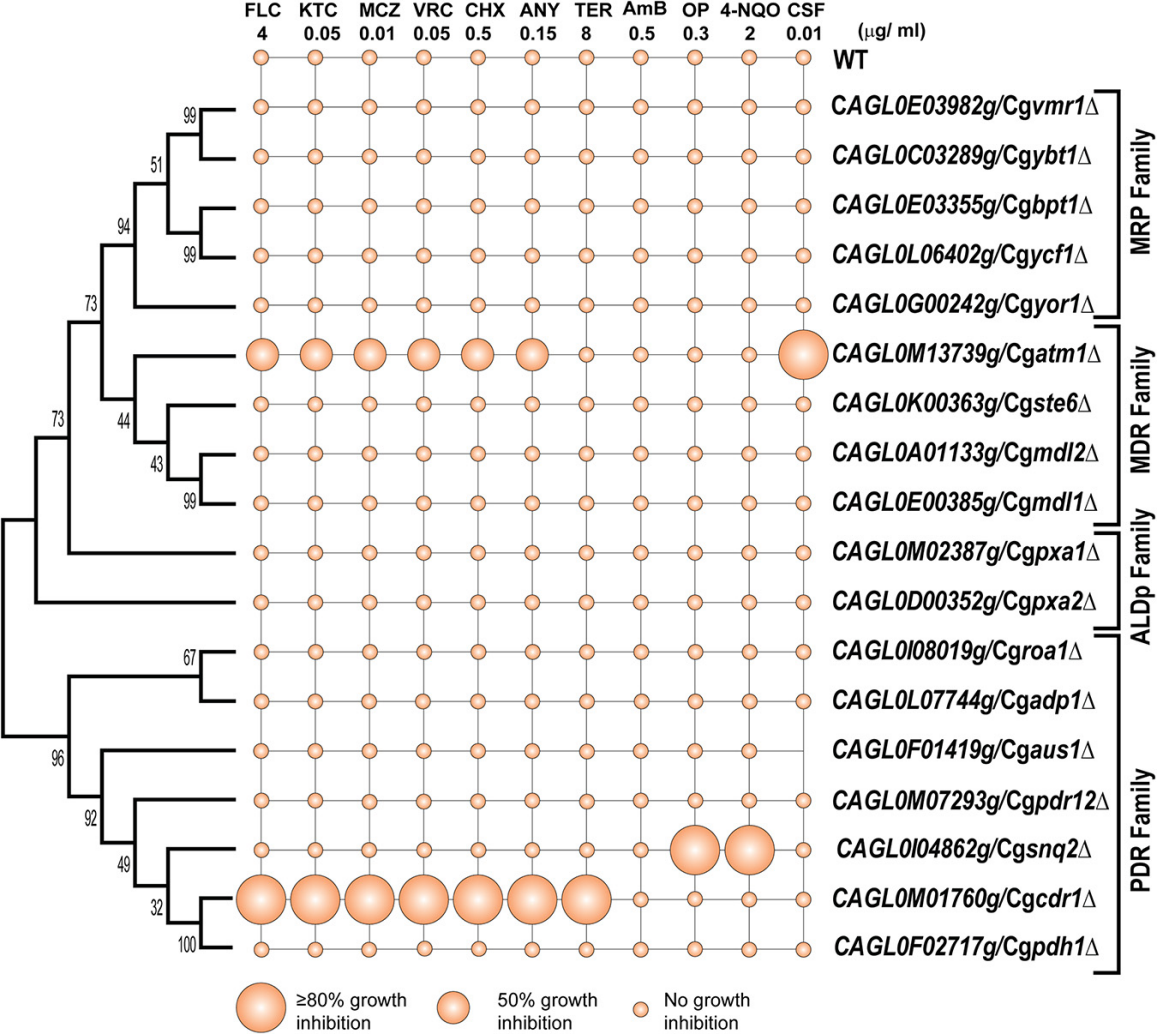
Phenotypic characterization of ABC transporter disruptome. Based on the MIC (MIC_80_), only 3 transporter deletion mutants are susceptible to different antimycotic drugs. A phylogenetic tree was constructed with ClustalW alignment of the complete protein sequence of 18 ABC transporters by MEGA7 with 1,000 bootstrap values.

The ABC transporter mutant library was screened for susceptibility toward different drugs (Table S3). Notably, the susceptibility to tested drugs remained unchanged in ABC transporter deletants belonging to the MRP and ALDp subfamilies. From the PDR subfamily, only Cg*cdr1*Δ and Cg*snq2*Δ manifested increased susceptibility to select drugs. As expected, Cg*cdr1*Δ cells are highly susceptible to antifungals belonging to the categories of azoles, allylamines, and protein synthesis inhibitors, thus confirming its predominant role as a significant multidrug transporter in C. glabrata (Fig. 1). Conversely, Cg*snq2*Δ was only susceptible to the DNA damaging agent 4-nitroquinoline N-oxide (4-NQO) and to the metal chelator 1,10-phenanthroline (OP), known substrates for its homolog ScSnq2 in S. cerevisiae (28, 29). The deletion of Cg*ATM1* from the MDR subfamily conferred susceptibility only to azoles and echinocandin caspofungin (CSF) and displayed no change concerning other drug exposure (Fig. 1).

10.1128/mbio.03545-21.7 TABLE S3 MIC of all ABC transporter mutants in the presence of different antifungal agents. Download Table S3, DOCX file, 0.2 MB. Copyright © 2022 Kumari et al. 2022 Kumari et al. https://creativecommons.org/licenses/by/4.0/ This content is distributed under the terms of the Creative Commons Attribution 4.0 International license.

Apart from Cg*cdr1*Δ, Cg*snq2*Δ, and Cg*atm1*Δ, all other transporter mutants remained unresponsive to the tested drug exposure, implying that individually, they have no significant role as drug transporters. However, considering the established role of some of the ABC transporters in clinical azole resistance in C. glabrata, we posit that their functions probably remain masked by the presence of dominant transporters such as CgCdr1.

### The dominant transporter CgCdr1 masks the function of other transporters.

**FIG 2:**
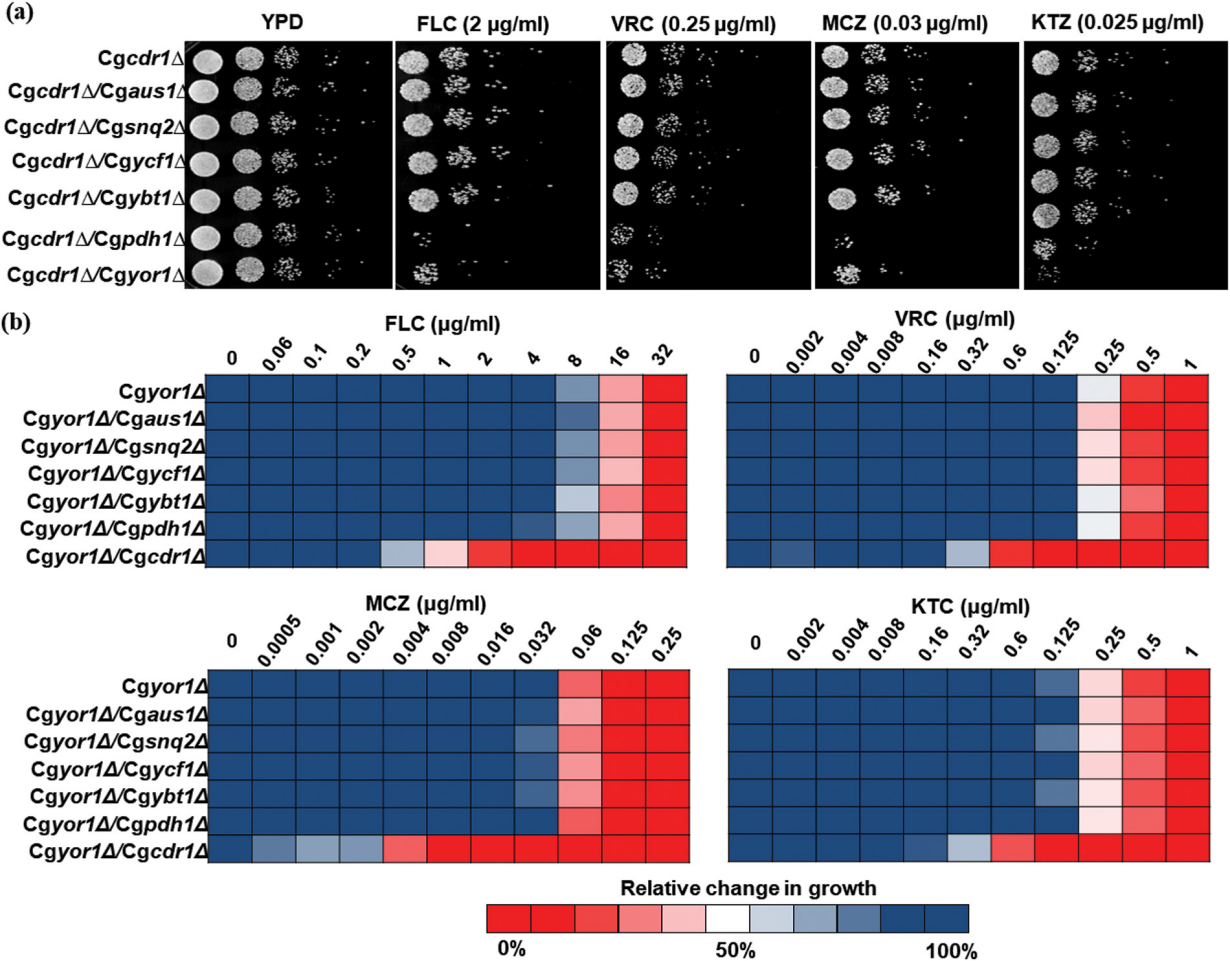
Susceptibility of ABC transporter mutants in the background of Cg*cdr1*Δ and Cg*yor1*Δ. Drug susceptibility to FLC, VRC, MCZ, and KTC was determined by (a) spot assays on solid agar media for mutants in the Cg*cdr1*Δ background and (b) broth microdilution in liquid media for mutants in the Cg*yor1*Δ deletion background.

ABC transporters share quite a high amino acid sequence homology and domain organization and have overlapping substrate profiles. For example, CgCdr1 shares a sequence identity of 72.49% with CgPdh1 and 48.19% with CgSnq2. This close similarity among ABC members demands more intense analysis to determine the contribution of each transporter toward the export of a substrate. The substrate specificity of S. cerevisiae ScPdr5, ScSnq2, and ScYor1 are overlapping (30, 31), which strongly suggests redundancy in their transport roles and the possibility of masking of individual function. Notwithstanding the reported dominant impact of CgCdr1 on CgPdh1 and CgSnq2 in C. glabrata (5, 15), the fact remains that there are studies pointing to the roles of many other ABC transporters (CgYor1, CgYcf1, and CgYbt1) in clinical azole resistance (18–21). To address this, we constructed double deletions of the transporter genes (Cg*PDH1*, Cg*SNQ2*, Cg*AUS1*, CgYCF1, Cg*YBT1*, and Cg*YOR1*) whose transcripts have been previously reported to be upregulated in drug-resistant clinical isolates of C. glabrata. Thus, each of such transporter-encoding genes was deleted in the Cg*cdr1*Δ background (Table S1). These double deletants, Cg*cdr1*Δ/Cg*pdh1*Δ, Cg*cdr1*Δ/Cg*snq2*Δ, Cg*cdr1*Δ/Cg*aus1*Δ, Cg*cdr1*Δ/Cg*yor1*Δ, Cg*cdr1*Δ/Cg*ycf1*Δ, and Cg*cdr1*Δ/Cg*ybt1*Δ, were then subjected to drug susceptibility profiling. The mutants Cg*cdr1*Δ/Cg*snq2*Δ, Cg*cdr1*Δ/Cg*aus1*Δ, Cg*cdr1*Δ/Cg*ycf1*Δ, and Cg*cdr1*Δ/Cg*ybt1*Δ exhibited no change in susceptibility to azoles compared to the Cg*cdr1Δ* strain, implying other cellular roles for these transporters independent from azole response (Fig. 2a). In contrast, Cg*cdr1*Δ/Cg*yor1*Δ and Cg*cdr1*Δ/Cg*pdh1*Δ showed increased susceptibility to azoles compared to Cg*cdr1*Δ (Fig. 2a), which was noticeably absent when these transporters were individually deleted (Table S3). Since the role of CgPdh1 in azole resistance is relatively well established (15), we focused this study on uncovering the role of CgYor1 in azole susceptibility, which remained masked in the presence of CgCdr1.

10.1128/mbio.03545-21.5 TABLE S1 Strains used in the study. Download Table S1, DOCX file, 0.02 MB. Copyright © 2022 Kumari et al. 2022 Kumari et al. https://creativecommons.org/licenses/by/4.0/ This content is distributed under the terms of the Creative Commons Attribution 4.0 International license.

The noticeably increased azole susceptibility in Cg*cdr1*Δ/Cg*yor1*Δ prompted us to explore if any other transporter could also conceal the influence of CgYor1 on azole susceptibility. To address this, we constructed double KOs in the background of Cg*yor1*Δ, such as Cg*yor1*Δ/Cg*pdh1*Δ, Cg*yor1*Δ/Cg*snq2*Δ, Cg*yor1*Δ/Cg*aus1*Δ, Cg*yor1*Δ/Cg*ycf1*Δ, and Cg*yor1*Δ/Cg*ybt1*Δ. We subjected each of these double KOs to drug susceptibility tests which also included the Cg*yor1*Δ/Cg*cdr1*Δ mutant. Significantly, none of the double KO mutants except Cg*cdr1*Δ/Cg*yor1*Δ could impact azole susceptibility (Fig. 2b). The data suggest that these transporters do not contribute to the masking of CgYor1 function.

### Cg*YOR1* deletion does not impart susceptibility to oligomycin.

**FIG 3:**
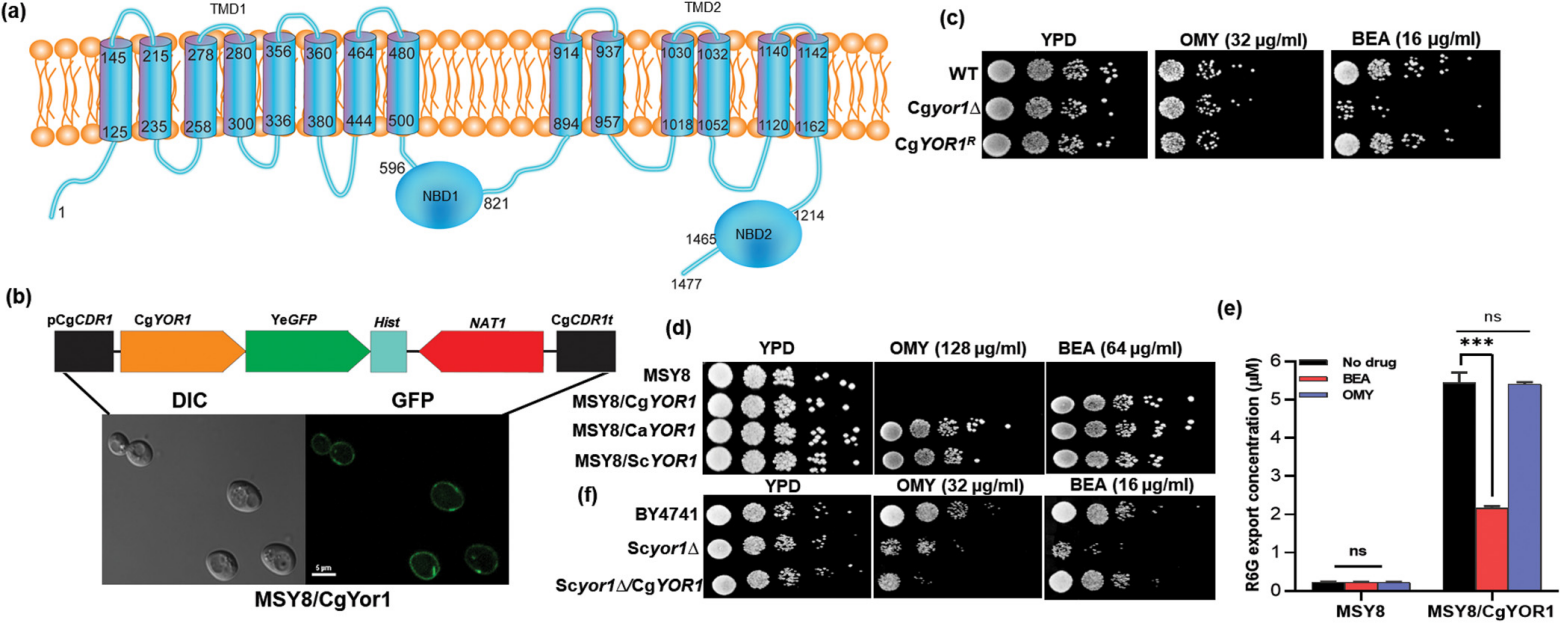
Topology, localization, and substrate specificity of CgYor1. (a) Predicted topology of CgYor1 generated by the softwares TOPOCONS and Scan Prosite results. (b) Localization of CgYor1 by expressing the Cg*YOR1* gene in the MSY8 strain at the Cg*CDR1* locus. (c) Spot assay drug susceptibility to OMY and BEA of the Cg*YOR1* WT, the deletion mutant (Cg*yor1*Δ), and revertant (Cg*YOR1^R^*). (d) Spot assay drug susceptibility to OMY and BEA of Cg*YOR1*, Ca*YOR1*, and Sc*YOR1* overexpressed in heterologous expression system MSY8 at the Cg*CDR1* locus. (e) Substrate competition; efflux of R6G by the MSY8^/^Cg*YOR1* overexpressing strain in the presence of 10× the MIC of BEA or OMY. (f) Complementation of S. cerevisiae *Scyor1*Δ with Cg*YOR1* and its susceptibility profile in the presence of OMY and BEA.

The topological prediction by TOPOCONS and Scan Prosite indicates that CgYor1 has a basic MRP subfamily topology, which consists of two transmembrane domains (TMDs) and two nucleotide binding domains (NBDs). TMD1 has eight transmembrane helixes (TMHs) followed by NBD1, while TMD2 has six TMHs followed by NBD2 (Fig. 3a). To characterize the localization of this transporter, we expressed the Cg*YOR1* gene in our homologous overexpression system, MSY8, which lacks 7 ABC transporters and has a GOF mutation of TF Cg*PDR1*. We integrated Cg*YOR1* at the hyperactive Cg*CDR1* locus as described earlier (27) and could confirm the overexpression of green fluorescent protein (GFP)-tagged CgYor1 protein by confocal images that showed a proper rimmed appearance at the plasma membrane (Fig. 3b). The properly overexpressed and localized CgYor1-GFP was fully functional, as was evident from its ability to show an elevated level of drug resistance and efflux of R6G, which was similar to the strain overexpressing untagged CgYor1 (Fig. S2a).

10.1128/mbio.03545-21.2 FIG S2 (a) Functionality of tagged and untagged CgYor1 overexpressed in MSY8. There was a similar BEA resistance profile of GFP-tagged CgYor1 as untagged CgYor1 and similar R6G efflux by both the tagged and untagged CgYor1 protein. (b) Synergism of FLC and BEA for the mutants indicated enhanced synergism between FLC and BEA in Cg*yor1*Δ. Download FIG S2, TIF file, 0.2 MB. Copyright © 2022 Kumari et al. 2022 Kumari et al. https://creativecommons.org/licenses/by/4.0/ This content is distributed under the terms of the Creative Commons Attribution 4.0 International license.

In both S. cerevisiae and C. albicans, Yor1 is known to transport organic anions, especially the macrolide oligomycin (OMY) (32, 33). A cyclic hexadepsipeptide natural product beauvericin (BEA) is another reported substrate of ScYor1 and CaYor1 (34, 35). Surprisingly, while Cg*yor1*Δ cells showed susceptibility to BEA, the susceptibility to OMY remained unchanged (Fig. 3c). We also tested the probability of enhanced efficacy of FLC and BEA combination treatment after Cg*yor1* deletion by employing a dose response matrix. There was greater synergy between FLC and BEA for Cg*yor1*Δ (fractional inhibitory concentration index [FICI] value, 0.093) than for WT (FICI value, 0.312), but the same was not evident for Cg*cdr*1*Δ* cells (FICI value, 1.50), which further supported that BEA is a substrate of CgYor1 (Fig. S2b). However, there was no synergy observed with OMY and FLC in both the mutants Cg*yor1*Δ and Cg*cdr1*Δ (data not shown). Further, we tested OMY and BEA susceptibility in the MSY8/Cg*YOR1* strain overexpressing the CgYor1 transporter. Notably, MSY8/Cg*YOR1* could not reverse susceptibility to OMY, while it could fully reverse susceptibility to BEA compared to MSY8. Expectedly, MSY8/Ca*YOR1* and MSY8/Sc*YOR1* strains reversed susceptibility to both OMY and BEA, suggesting these to be substrates of CaYor1 and ScYor1 (Fig. 3d).

Moreover, substrate competition assays of R6G efflux in the presence of 10× the MIC of either OMY or BEA in the MSY8/Cg*YOR1* strain further provided credence to our observation. The efflux of R6G by CgYor1 was reduced by half in the presence of BEA but remained unchanged in the presence of OMY (Fig. 3e). To further confirm that OMY is not a substrate of CgYor1, we complemented the Sc*yor1Δ* strain with the Cg*YOR1* gene and checked its phenotype with respect to OMY and BEA. Notably, the complementation with CgYor1 did not change the Sc*yor1Δ* strain susceptibility to OMY but did reverse the susceptibility to BEA. These results again strengthened our hypothesis that OMY may not be a substrate of CgYor1 (Fig. 3f).

### Azoles are not the substrate of CgYor1.

**FIG 4:**
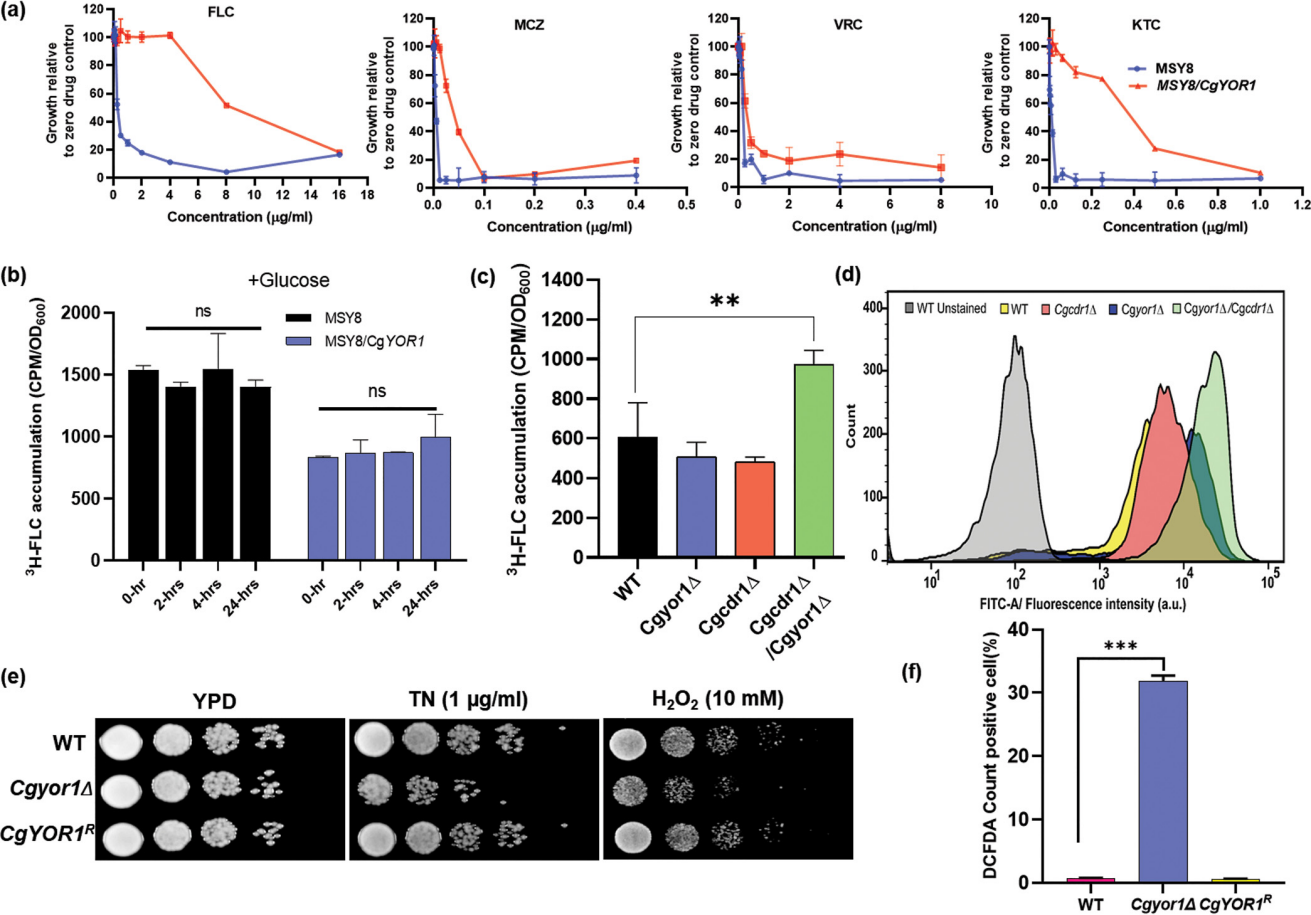
Analysis of factors behind the azole phenotypes of CgYor1. (a) Overexpression of CgYOR1 in MSY8 and phenotypic characterization of azole susceptibility by MIC analysis. (b) ^3^H-FLC accumulation in MSY8 and MSY8/Cg*YOR1* strains in both the presence and absence of energy. (c) Accumulation of ^3^H-FLC in the absence of energy to examine the diffusion rate in the Cg*yor1*Δ, Cg*cdr1*Δ, and Cg*cdr1*Δ/Cg*yor1*Δ deletion. (d) R6G accumulation in the strains Cg*yor1*Δ, Cg*cdr1*Δ, and Cg*cdr1*Δ/Cg*yor1*Δ in order to confirm the change in the diffusion of substrates. (e) Cg*yor1*Δ susceptibility to TN and H_2_O_2_ by spot analysis displayed increased ER stress and endogenous ROS generation. (f) ROS measurement by FACS analysis using DCFDA in the WT, Cg*yor1Δ*, and Cg*YOR1^R^* strains.

Based on the fact that the Cg*cdr1*Δ/Cg*yor1*Δ strain is susceptible to azoles, we suspected that azoles could be the substrate of CgYor1. To test this, we measured azole susceptibility in the MSY8/Cg*YOR1* cells. We noticed the reversal of susceptibility to all the tested azoles in the cells overexpressing CgYor1 (Fig. 4a). To determine whether FLC is the substrate of CgYor1, we performed a time course study of ^3^H-FLC accumulation in MSY8/Cg*YOR1* cells as detailed in Materials and Methods. Cells were preloaded with ^3^H-FLC for 24 h without an energy source, and then 2% glucose was provided as an energy source to activate efflux transporters. Time course analysis of intracellular ^3^H-FLC levels was performed over 24 h upon glucose addition. Intracellular ^3^H-FLC levels in MSY8/Cg*YOR1* cells at every time point of sample collection showed no reduction, implying that FLC is not transported by CgYor1 (Fig. 4b). To further exclude the possibility of CgYor1 as a transporter of FLC, we also investigated substrate competition with R6G using FLC as the competitive substrate in the MSY8/CgYOR1 strain. We observed that at concentrations of FLC up to 10× MIC, FLC could not compete with R6G efflux, implying that the FLC is not a substrate of CgYor1 (Fig. S3a). Under a similar set of conditions, BEA, which is a known substrate CgYor1, did compete with R6G efflux (Fig. 3e).

10.1128/mbio.03545-21.3 FIG S3 (a) R6G efflux with FLC competition in MSY8 and MSY8/*YOR1*. (b) ^3^H-FLC accumulation after 24 h of incubation without an energy source in MSY8/Cg*YOR1*. (c) Ergosterol level in Cg*yor1*Δ, Cg*cdr1*Δ, and Cg*cdr1*Δ/Cg*yor1*Δ strains. (c) Transcript level expression of ERG genes in the Cg*yor1*Δ mutant. (d) R6G substrate competition with FK520 and GA in MSY8 and MSY8/*YOR1.* Download FIG S3, TIF file, 0.2 MB. Copyright © 2022 Kumari et al. 2022 Kumari et al. https://creativecommons.org/licenses/by/4.0/ This content is distributed under the terms of the Creative Commons Attribution 4.0 International license.

### Cg*YOR1* deletion in Cg*cdr1*Δ confers enhanced FLC diffusion.

The intracellular ^3^H-FLC accumulation was monitored by incubating the cells with ^3^H-FLC for 24 h after energy deprivation for 3 h to inhibit the effect of ATP-dependent efflux, as described in Materials and Methods. In the absence of energy, ABC transporters become inactive and the accumulation of ^3^H-FLC depends on the facilitated diffusion through membrane in fungi (36). We observed reduced ^3^H-FLC accumulation in MSY8/Cg*YOR*1 cells after the preloading period of 24 h without an energy source compared to the MSY8 strain (Fig. S3b). We speculate that if the overexpression of Cg*YOR1* results in reduced azole accumulation in MSY8/Cg*YOR*1 cells, then the deletion of Cg*YOR1* could increase azole accumulation and susceptibility in Cg*yor1Δ* cells.

We also explored the differences in passive diffusion of FLC, which could explain the increased susceptibility displayed by Cg*cdr1*Δ*/*Cg*yor1*Δ cells. The observation obtained from the accumulation analysis of these strains indicates that the double deletion strain Cg*cdr1*Δ/Cg*yor1*Δ accumulates a larger amount of ^3^H-FLC than the WT strain as well as the single deletion strains Cg*cdr1*Δ and Cg*yor1*Δ (Fig. 4c). This suggests that the combined deletion of these two membrane transporter genes has dramatically affected the membrane of the strain. The increased accumulation of ^3^H-FLC after these two gene deletions could be a reason for the enhanced azole susceptibility in the double mutant.

To further confirm that the membrane of the double mutant is compromised and there is a change in the diffusion rate, we performed the accumulation of R6G in these mutant cells. Cells were initially deprived of energy for 3 h and then incubated with R6G for 45 min. The fluorescence-activated cell sorter FACS analyses with these mutants resembled the results with ^3^H-FLC. The Cg*cdr1*Δ/Cg*yor1*Δ mutant displayed a higher accumulation of R6G than the other strains (Fig. 4d). These results suggest a change in membrane diffusion properties in the Cg*cdr1*Δ/Cg*yor1*Δ cells may lead to a higher accumulation of FLC inside the cells.

### Cg*YOR1*Δ leads to ER stress and enhanced ROS generation.

Azoles target ER-localized Erg11 and block the production of ergosterol, which leads to ergosterol depletion from the plasma membrane and the accumulation of toxic sterol intermediates in the cell (37). Independent studies in several pathogenic fungi point to a role of ER stress in influencing antifungal resistance and pathogenesis (38). ER stress has been shown to enhance the effect of azoles and show synergy with the antifungals that lead to a defect in protein folding, Ca2+ homeostasis, and ROS generation (38, 39). Azoles increase reactive oxygen species (ROS) generation in *Candida* and in other fungal species cells, albeit to different magnitudes (40–42).

First, we checked if the targets of azoles are being affected in Cg*yor1Δ* cells. In this context, the transcript levels of Cg*ERG11*, Cg*ERG3*, Cg*ERG6*, Cg*ERG4*, and Cg*ERG2* in Cg*yor1Δ* cells were assessed by quantitative PCR (qPCR). As evident from Fig. S3d, there was no significant change in *ERG* gene expression. Similarly, spectrometric-based analysis of ergosterol in the mutants also showed no changes in the ergosterol content (Fig. S3c). These data confirmed that Cg*yor1*Δ cells do not have any major change in the azole target gene and ergosterol content.

The impact of Cg*YOR1* deletion on ER stress was examined by exposing Cg*yor1Δ* cells to a well-known ER stress inducer, tunicamycin (TN), which is shown to block the ER enzyme UDP-*N*-acetylglucosamine-1-P transferase required for N-glycosylation in S. cerevisiae cells (43). The Cg*yor1Δ* mutant displayed enhanced susceptibility to TN, which was rescued in Cg*YOR1^R^* revertant cells (Fig. 4e). This pointed to an increased ER stress in Cg*yor1*Δ cells as was also reported in Sc*yor1*Δ cells (44). The deletion of Cg*YOR1* also conferred increased susceptibility to H_2_O_2,_ indicating defective oxidative stress management (Fig. 4e). Further, we checked the status of endogenous ROS production in the mutant Cg*yor1Δ* cells. ROS was quantitated by employing an oxidant-sensitive fluorescent probe 2′,7′-dichlorofluorescein diacetate (DCFDA), where its fluorescence intensity is directly proportional to ROS levels (45). By employing FACS analysis, we observed that 30% more Cg*yor1*Δ cells were taking up DCFDA dye than WT cells and Cg*YOR1^R^* revertant cells (Fig. 4f). Together, the observed ER stress and ROS generation in the Cg*yor1*Δ cells prompted us to explore if these multiple effects can influence the stress-related signaling cascades that could influence azole susceptibility.

### Cg*yor1Δ* mutants display enhanced azole susceptibility under nitrogen depletion conditions.

Nitrogen is an essential nutrient for yeast growth, and its availability or scarcity has numerous consequences on gene expression, signaling, and metabolism (46). Nitrogen starvation could have an impact on SNF1/AMP-activated protein kinase (AMPK), cAMP-dependent protein kinase (PKA), and TOR signaling pathways that leads to substantial transcriptomic changes (47). It is established that nitrogen starvation suppresses TOR kinases in several yeasts (48). To assess the impact of nitrogen starvation, we grew the WT strain, the Cg*yor1*Δ strain, and its revertant Cg*YOR1^R^* under both nitrogen-replete (N-replete) and nitrogen-depleted (N-depleted) conditions as detailed in Materials and Methods.

**FIG 5:**
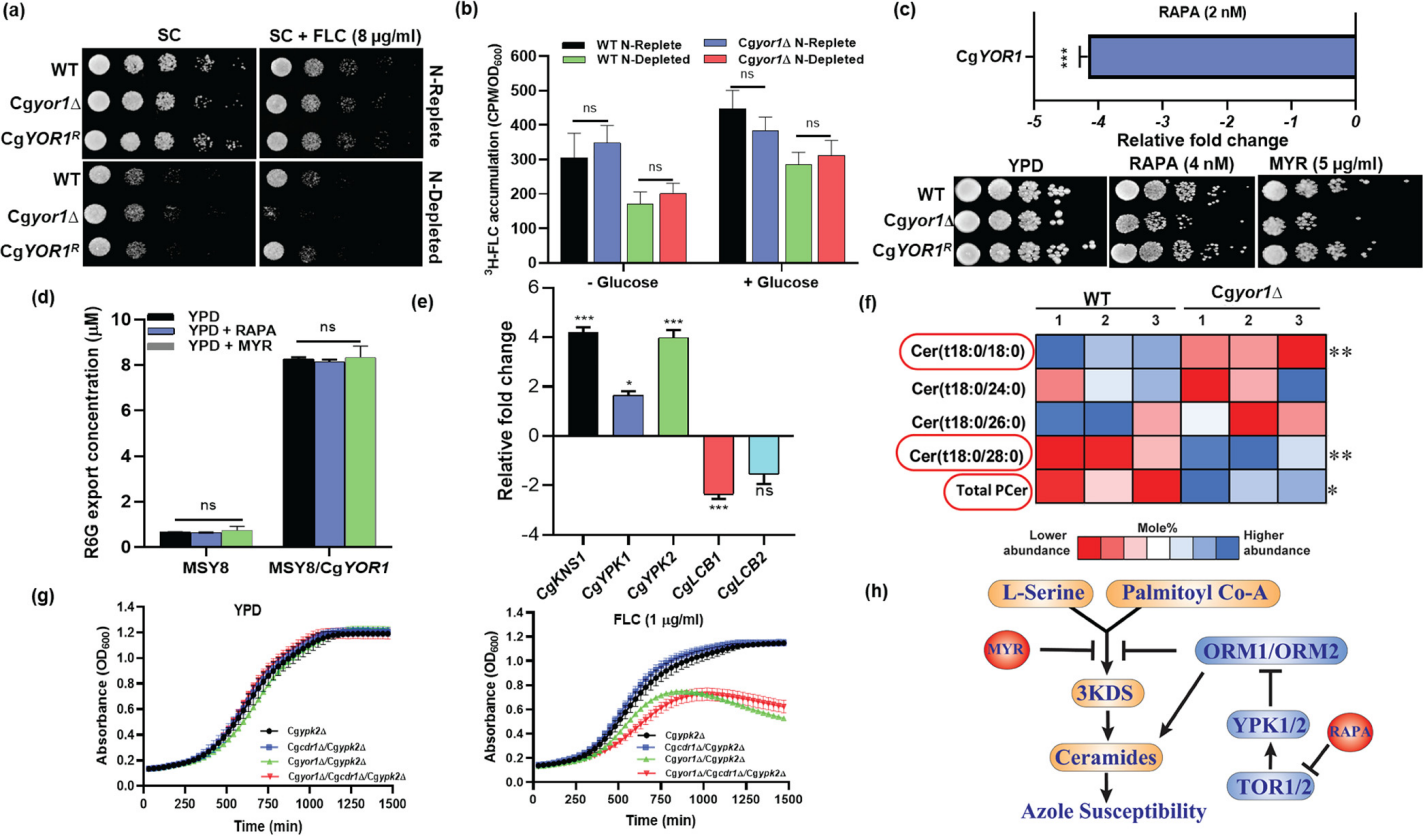
Cg*YOR1* deletion led to suppressed TOR kinase. (a) To assess the impact of nitrogen availability, the WT, Cg*yor1*Δ, and revertant Cg*YOR1^R^* strains were grown under N-replete and N-depleted conditions. Cells were then spotted on SC agar containing FLC. (b) ^3^H-FLC accumulation under both N-depleted and N-replete conditions in the presence and absence of energy source. (c) Susceptibility of the WT, Cg*yor1*Δ, and Cg*YOR1^R^* strains in RAPA and MYR by spot analysis and transcript-level expression of Cg*YOR1* in the WT strain after transient treatment with RAPA. (d) Substrate competition of RAPA and MYR with R6G. (e) Transcript level expression of TOR kinase downstream effector genes in Cg*yor1*Δ. (f) PCer level comparison in WT and Cg*yor1*Δ. P-Cers with significant differences in abundance are circled in red. 1, 2, and 3 indicate data from triplicate experiments. (g) Growth curves of Cg*ypk2*Δ, Cg*cdr1*Δ/Cg*ypk2*Δ, Cg*yor1*Δ/Cg*ypk2*Δ, and Cg*yor1*Δ/Cg*cdr1*Δ/Cg*ypk2*Δ strains when grown in YPD or YPD +FLC. (h) Schematic displaying the impact of TOR kinase on ceramide level and azole susceptibility.

In agreement with the azole susceptibility data in Fig. 2b, Cg*yor1*Δ single deletion does not alter azole susceptibility compared to the WT strain in N-replete conditions (Fig. 5a). The WT, Cg*Yor1Δ*, and Cg*YOR1^R^* revertant strains were all affected equally by treatment with fluconazole when nitrogen was present. However, after 12 h of growth in N-depleted conditions, the WT, Cg*Yor1Δ*, and Cg*YOR1*^R^ revertant strains had reduced growth on agar and grew poorly in liquid media. Addition of FLC further inhibited growth, and FLC susceptibility was more pronounced for all strains under N-depleted conditions (Fig. 5a). This was particularly evident in the Cg*yor1*Δ strain, which showed enhanced susceptibility to FLC under N-depleted conditions. This FLC susceptibility could be rescued to WT levels in Cg*YOR1^R^* cells (Fig. 4a). Noticeably, Cg*cdr1*Δ cells displayed a similar level of susceptibility to FLC under both N-depleted and N-replete conditions (Fig. S4a).

10.1128/mbio.03545-21.4 FIG S4 (a) Cg*cdr1*Δ phenotype on N-depleted and N-replete media. (b) Transcript level expression of Cg*CDR1* in Cgyor1 deletion under N-replete and N-depleted conditions. (c) TOR down-effector genes and calcineurin subunit (Cg*CNB1*) deletion and in Cg*yor1*Δ, Cg*cdr1*Δ deletion, and double deletion of Cg*cdr1*Δ/Cg*yor*1Δ growth curve analysis in the presence of FLC. Download FIG S4, TIF file, 0.4 MB. Copyright © 2022 Kumari et al. 2022 Kumari et al. https://creativecommons.org/licenses/by/4.0/ This content is distributed under the terms of the Creative Commons Attribution 4.0 International license.

In Cg*yor1*Δ cells grown under N-replete conditions, the transcript of Cg*CDR1* was increased by 2.5-fold (Fig. S4b), which might explain why the *Cgyor1*Δ strain is not susceptible to FLC in the N-replete conditions (Fig. 5a). But under N-depleted conditions, the transcript of Cg*CDR1* was downregulated 4-fold in the Cg*yor1*Δ cells (Fig. S4b). These data imply that CgCdr1 is unable to mask the azole susceptibility phenotype of the Cg*yor1*Δ cells under N-depleted conditions. The change in Cg*CDR1* transcription levels in response to the presence or absence of nitrogen does not affect FLC accumulation into the cells. Under both N-replete and N-depleted conditions, intracellular accumulation of ^3^H-FLC was very similar in the presence or absence of an energy source (Fig. 5b). Together, these data establish the masking of the CgYor1 function by CgCdr1 under normal growth conditions. The data also support the fact that the azole susceptibility in Cg*yor1*Δ mutant cells may involve the TOR kinases.

### Lack of Cg*YOR1* suppresses TOR signaling.

TOR stands for “target of rapamycin” and refers to conserved serine/threonine kinases that control cell growth in response to nutrients (49). In C. glabrata two paralogs of TOR kinase (TOR1 and TOR2) exist, which form two structurally and functionally distinct complexes, TOR complex 1 (TORC1) and TOR complex 2 (TORC2) (50). TOR1 is a rapamycin-sensitive kinase that controls cell growth in response to nutrients by promoting anabolic processes such as translation and ribosome biogenesis, while TOR2 is mainly regulated in response to changes in sphingolipid metabolism (51, 52). In C. albicans and S. cerevisiae, suppression of TOR kinases potentiates the activity of antifungal agents (34).

Involvement of the TOR pathway in the Cg*yor1*Δ phenotype was assayed by comparing the growth of WT, Cg*yor1*Δ, and Cg*YOR1^R^* cells in the presence of rapamycin (RAPA), which targets TOR kinase and myriocin (MYR), which acts on the serine-palmitoyltransferase (SPT) complex affecting the sphingolipid homeostasis. We also demonstrated an increased susceptibility of FLC in the Cg*yor1*Δ cells under nitrogen-depleted conditions, which is also an indicator of TOR-suppressed conditions (53, 54). The Cg*yor1*Δ mutant displayed increased susceptibility on both RAPA and MYR, and this susceptibility was rescued in the revertant Cg*YOR1^R^* cells (Fig. 5c). Noticeably, the transcript level of Cg*YOR1* was 4-fold downregulated when WT cells were exposed to RAPA (Fig. 5c), while no change was observed in transcripts levels of the other six ABC transporter-encoding genes (Cg*CDR1*, Cg*PDH1*, Cg*SNQ2*, Cg*AUS1*, Cg*YBT1*, and Cg*CYCF1*) (data not shown). To exclude the possibility that CgYor1 could transport RAPA or MYR, we performed a substrate competition efflux assay with R6G in the presence of 10× the MIC of RAPA or MYR in the MSY8/Cg*YOR1* strain. The R6G efflux activity remained unchanged in the presence of excess RAPA and MYR in MSY8/Cg*YOR1* cells, thus ruling out the possibility of RAPA and MYR being substrates of CgYor1 (Fig. 5d).

The involvement of TOR kinase in suppressing the growth of the Cg*yor1*Δ mutant in the presence of FLC was also validated by monitoring transcript levels of the downstream genes (Cg*KNS1*, Cg*YPK1*, Cg*YPK2*, Cg*LCB1*, and Cg*LCB2*) involved in the signaling cascade. *KNS1* is negatively regulated by TOR1 kinase in yeast, and interestingly, the transcript of Cg*KNS1* was upregulated in Cg*yor1*Δ cells, consistent with the suppression of TOR1 in Cg*yor1*Δ mutant cells. Moreover, downregulation of both SPT enzyme-encoding genes, Cg*LCB1* and Cg*LCB2*, was observed in Cg*yor1*Δ, which suggests interference of TOR2 kinase (Fig. 5c and e). This is consistent with MYR susceptibility, which blocks SPT enzymes.TOR1 and TOR2 interact with several downstream effector kinases such as Orm1, Orm2, Ypk1, and Ypk2 in yeast (55). In Cg*yor1*Δ, inactivation of TOR2 kinases activates CgYpk1 and CgYpk2, which can also be seen through their transcript-level expression. The upregulation of Cg*YPK1* and Cg*YPK2* in Cg*yor1*Δ further supports the involvement of TOR kinases (Fig. 5e). Additionally, the deletion of effector kinases (Cg*ckb1*Δ, Cg*ckb2*Δ, Cg*ypk1*Δ, Cg*ypk2*Δ, Cg*cnb1*Δ) in the Cg*yor1*Δ or Cg*yor1*Δ/Cg*cdr1*Δ strain background and their phenotypic analysis in the presence of FLC supported the conclusions.

The susceptibility to MYR and transcript level change in Cg*YPK1* and Cg*YPK2* indicate the involvement of sphingolipids, which are important membrane lipid biomolecules known to regulate various stress-related signaling pathways (56). The sphingolipid profiles of the WT and Cg*yor1*Δ strains were determined by liquid chromatography tandem mass spectrometry (LC-MS/MS)-based analysis. Interestingly, the phytoceramide (PCer) level, which is the most abundant ceramide and can influence azole resistance (57), was significantly increased in the Cg*yor1*Δ mutant (Fig. 5f). A significant increase in Cer(t18:0/28:0) was observed in Cg*yor1*Δ cells, indicating that the increased PCer level could be influencing azole susceptibility. Cer(t18:0/18:0) also displayed significant differences between the WT and Cg*yor1Δ* strains. However, no change was observed in the rest of the major sphingolipids, such as dihydroceramide, α-hydroxyceramides, and others (Table S4).

10.1128/mbio.03545-21.8 TABLE S4 Sphingolipid profile of the mutants (data are represented as the average of mole% values of biological triplicates, and values shown in bold are significant changes with respect to WT). Download Table S4, DOCX file, 0.02 MB. Copyright © 2022 Kumari et al. 2022 Kumari et al. https://creativecommons.org/licenses/by/4.0/ This content is distributed under the terms of the Creative Commons Attribution 4.0 International license.

Ceramide levels can be influenced through TOR2 and regulated by CgYpk1 and CgYpk2. CgYpk1 and CgYpk2 work downstream of TOR2 to regulate the ceramide level (58). To address the effect of CgYpk1 and CgYpk2 on azole resistance in the Cg*yor1*Δ strain, we have constructed the double mutants Cg*ypk1*Δ/Cg*yor1*Δ and Cg*ypk2*Δ/Cg*yor1*Δ. Both deletions enhanced the FLC susceptibility of Cg*yor1*Δ; however, the effect of Cg*ypk2*Δ in Cg*yor1*Δ was greater. Importantly, the deletion of Cg*YPK1* and Cg*YPK2* in Cg*cdr1*Δ did not display any further enhanced susceptibility to FLC (Fig. 5g and Fig. S4c). Similarly, deletion strains Cg*orm1*Δ and Cg*orm2*Δ in the Cg*yor1*Δ deletion strain also displayed slightly increased susceptibility, pointing to TOR kinase involvement in the CgYor1 effect on azole susceptibility (Fig. S4c). Together, the data point to the influence of TOR kinases on azole susceptibility by influencing the PCer level through their effector kinases (CgYpk1, CgOrm1, CgOrm2, and especially, CgYpk2) in Cgyor1Δ (Fig. 5h). However, to establish a direct connection between ceramide levels and downstream kinases, further analysis is required.

### The absence of CgYor1 suppresses the calcineurin pathway.

**FIG 6:**
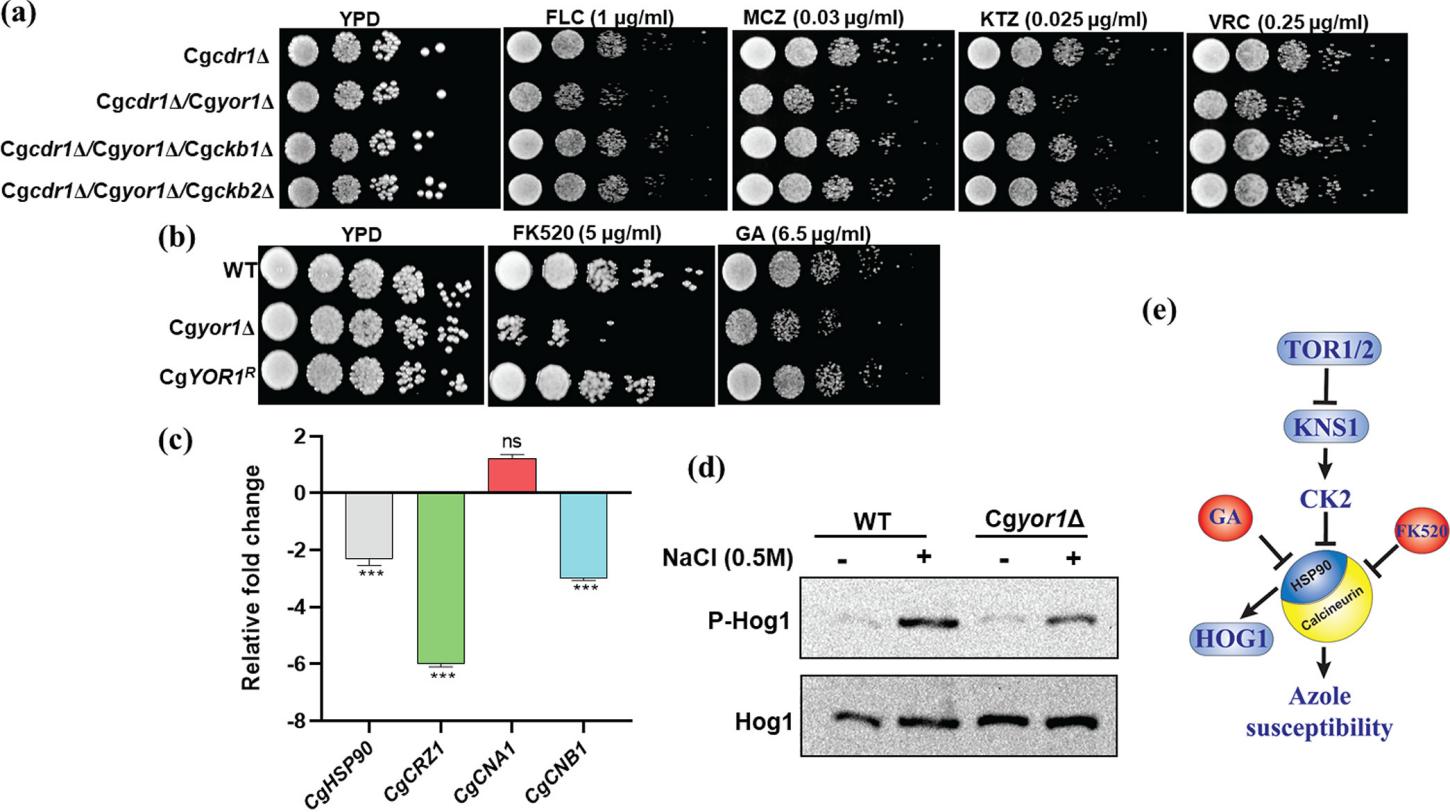
Calcineurin is suppressed in Cg*yor1*Δ. (a) Deletion of the regulatory subunits (CgCkb1 and CgCkb2) of protein kinase CK2 reverses the azole-susceptible phenotype of Cg*cdr1*Δ/Cg*yor1*Δ. (b) Cg*yor1*Δ is susceptible to the calcineurin inhibitor FK520 and Hsp90 inhibitor geldanamycin (GA). (c) Transcript level expression of CgHSP90, calcineurin subunits Cg*CNA*1 and Cg*CNB*1, and the transcription factor-encoding gene CgCRZ1 in Cg*yor1*Δ relative to WT. (d) Phosphorylation of CgHog1 in the presence and absence of salt stress in the WT and Cg*yor1*Δ strains. (e) Pathway displaying the involvement in TOR and calcineurin in azole resistance.

Calcineurin is a mediator of various stress responses and plays a critical role in mediating drug resistance and virulence in various fungi, including C. glabrata (59, 60). Calcineurin, a serine-threonine-specific protein phosphatase, is stabilized by molecular chaperone Hsp90. Hsp90 is phosphorylated by protein kinase CK2, which is a tetramer of four subunits—two activator α-subunits (Cka1 and Cka2) and two regulatory β-subunits (Ckb1 and Ckb2).The KNS1 kinase phosphorylates regulatory subunit CKb2 of CK2, resulting in its activation (61). The active phosphorylated CK2, in turn, phosphorylates Hsp90 and suppresses the calcineurin, which makes cells unable to combat various stresses, including azoles (62, 63). A previous report has indicated that both the regulatory and activation subunits of protein kinase CK2 mutants are involved in azole resistance in C. albicans (64). To check whether CK2 is activated in the Cg*cdr1*Δ/Cg*yor1*Δ mutant and has any effect on azole resistance, we constructed the regulatory subunit KO strain in the background of Cg*cdr1*Δ/Cg*yor1*Δ (Cg*cdr1*Δ/Cg*yor1*Δ/Cg*ckb1*Δ, and Cg*cdr1*Δ/Cg*yor1*Δ/Cg*ckb2*Δ). We observed that Cg*cdr1*Δ/Cg*yor1*Δ harboring any of the regulatory subunit deletions displays reversal of azole susceptibility, implying the effect of protein kinase CK2 on CgYor1-mediated azole susceptibility (Fig. 6a).

The suppression of TOR kinase causes suppression of Hsp90 activity, which destabilizes the protein phosphatase calcineurin, resulting in its deactivation (65, 66). The suppression of CgHsp90 by CK2 activation was also evident with its 2-fold downregulated transcript level in Cg*yor1*Δ cells along with downregulated calcineurin regulatory subunit transcript (Cg*CNB1*) (Fig. 6c). The suppressed calcineurin signaling cascade in the Cg*yor1*Δ mutant was further confirmed when we tested calcineurin inhibitor FK520 susceptibility. We observed that Cg*yor1*Δ mutant cells showed increased susceptibility to FK520. Similarly, we have observed an enhanced susceptibility of the Cg*yor1*Δ mutant toward geldanamycin (GA), an inhibitor of Hsp90. Both FK520 and GA susceptibility are not due to the efflux activity of CgYor1, which was confirmed by their competition with R6G in the MSY8/CgYOR1 strain (Fig. S3e). The susceptibility of the mutant to GA suggests suppressed CgHsp90 in the Cg*yor1*Δ, resulting in unstable calcineurin (Fig. 6b). To further confirm it, we have analyzed the phosphorylation level in CgHog1, which is a mitogen-activated protein kinase (MAPK) of the high osmotic glycerol pathway and is activated under osmotic stress (50). Hsp90 affects activation of Hog1 through its phosphorylation (67). We did not observe CgHog1 phosphorylation in the WT and Cg*yor1*Δ mutant under normal conditions; however, hypo-phosphorylation of CgHog1 became evident in the Cg*yor1*Δ mutant after treatment with salt stress (0.5 M NaCl), which was used to activate CgHog1 phosphorylation (Fig. 6d). The hypo-phosphorylation of CgHog1 in the Cg*yor1*Δ strain confirms less active CgHsp90 in Cg*yor1*Δ mutant cells.

To further investigate the effect of unstable Hsp90 and calcineurin, we constructed a deletion of the regulatory subunit of calcineurin Cg*CNB1*, whose transcript was also downregulated in the Cg*yor1*Δ strain. The Cg*cnb1*Δ in the Cg*yor1*Δ mutant demonstrated a slight increase in susceptibility toward FLC, which indicated that calcineurin is affected in the Cg*yor1*Δ mutant (Fig. S4c). Of note, the zinc finger TF CgCrz1 regulates calcineurin function. A 6-fold downregulation of the Cg*CRZ1* transcript in the Cg*yor1*Δ mutant cells further support the link between suppressed calcineurin and azole susceptibility (Fig. 6c). Overall, our results suggest that in the absence of CgYor1, calcineurin protein was suppressed due to hyperactive CK2 and less active Hsp90, thus contributing to azole susceptibility (Fig. 6e).

## DISCUSSION

Candida cells harbor a battery of ABC transporters in their genome, and only a few of them have been implicated in clinical drug resistance. The abundance of ABC transporters in *Candida* strongly hints at their multiple roles. This assertion is well supported by several independent observations. For instance, ABC transporters, apart from being drug transporters, are also shown to govern membrane lipid translocation, endocytosis, maintenance of mitochondrial integrity, and many additional virulence traits. (68, 69). ABC transporters such as ScPdr5, CaCdr1, CaCdr2, and CaCdr3 are phospholipid translocators that maintain membrane asymmetry. MRP subfamily transporters (Yor1, Ybt1, Ycf1, Mlt1, and Bpt1) in diverse fungi are involved in the transport of phosphatidylcholine (PC), phosphatidylethanolamine (PE), ions, bile salts, and vacuolar detoxification (70–73). CnAfr1 of C. neoformans and AbcB of Aspergillus fumigatus are required for pathogenesis (74, 75). Biofilms of *Candida* spp. demonstrate an upregulation of ABC transporters, indicating their potential role in biofilm formation and resistance to antifungals (76–79).

While multiple roles of ABC transporters are becoming increasingly clear, the masking effect due to redundancy and overlap in function, compensatory changes in expression in response to stimulus, the presence of major drug transporters, and other processes may obscure the functional relevance of specific transporters. We addressed this issue using haploid C. glabrata, which is one of the most common pathogenic *Candida* spp., and created a library of single deletion mutants of all 18 ABC membrane proteins to uncover the function of these transporters. While the phenotypes of our deletion library strains confirm the role of CgCdr1 as a major azole drug transporter in C. glabrata, we also discovered that CgYor1 contributes to azole resistance, while it remains masked by CgCdr1. Thus, the role of CgYor1 in azole resistance was only evident in the background of Cg*cdr1*Δ. Notably, besides CgCdr1, no other transporter single or double deletion could unmask the function of CgYor1. We also discovered that unlike the designated function of Yor1 as an OMY transporter in different yeasts, CgYor1 neither impacts susceptibility toward OMY nor transports it. However, as is observed for Yor1 of other yeasts, BEA remains a substrate of CgYor1. The inability of CgYor1 to export OMY is consistent with its failure to complement Sc*yor1*Δ in restoring OMY resistance. The substrate competition studies showing efflux of the test substrate R6G could be competed out by BEA and not by OMY provide further credence to our observation that CgYor1, unlike in other yeasts, does not transport OMY.

A report of an azole-resistant clinical isolate of C. glabrata demonstrated a drastic decrease of FLC MIC from 256 μg/mL to as low as 2 μg/mL upon deletion of the TF Cg*PDR1*. However, single deletion of Cg*CDR1* in the same azole-resistant isolate only reduced the FLC MIC to 16 μg/mL. Triple deletions of Cg*CDR1*, Cg*PDH1*, and Cg*SNQ2* resulted in the FLC MIC decreasing to 8 μg/mL (5). These observations strongly suggest that additional undiscovered transporters could contribute to drug resistance in C. glabrata. CgYor1 transporter is also part of the regulon of TF CgPdr1 in C. glabrata; however, the contribution of CgYor1 in clinical antifungal resistance has not been well defined (10). In this study, we have observed that the azole susceptibility is reversed in the MSY8 strain overexpressing Cg*YOR1*; however, ^3^H-FLC and efflux data suggest the reversal of azole susceptibility is not linked to FLC transport by CgYor1.

Lipids are a major constituent of plasma membrane and help in maintaining the membrane integrity (80). CgYor1 could be important to maintain membrane lipid homeostasis, which was evident from the altered sphingolipid composition in Cg*yor1*Δ and could be a reason for the increased passive or facilitated diffusion of the ^3^H-FLC and fluorescent dye R6G in the Cg*cdr1*Δ/Cg*yor1*Δ. The increased intracellular accumulation of FLC could lead to several physiological consequences, as was evident from an increased ROS level and enhanced ER stress in the Cg*yor1*Δ mutant. These changes were suggestive of the involvement of other downstream signaling pathways. For instance, TOR kinases, calcineurin, and their downstream signaling partners are shown to influence azole resistance in C. albicans and S. cerevisiae (34, 35). Our observations demonstrate that both TOR1 and TOR2 kinases and their downstream signaling partners have important roles in regulating CgYor1-mediated azole resistance. A direct link between CgYor1 and TOR kinases was evident from the fact that the deletion of Cg*YOR1* combined with nitrogen depletion independently suppresses TOR signaling, resulting in increased susceptibility to azoles. Deletion of Cg*CDR1* under nitrogen-depleted and -replete conditions had a similar impact on azole susceptibility, suggesting the independent function of CgYor1 from CgCdr1.

Several studies highlight the signaling role of sphingolipid molecules in multiple cellular processes, including azole resistance (81–83). Sphingolipid long-chain bases (LCBs) are the key intermediates in sphingolipid biosynthesis that include dihydrosphingosine (DHS), phytosphingosine (PHS), and their phosphorylated forms (84). In C. albicans azole stress can induce LCB formation where PHS-1-P seems to play an important role in azole resistance by mediating azole accumulation in cells (85). The ceramides are also important as signal molecules that modulate several biochemical and cellular responses to stress stimuli (56, 86). Ceramide and sphingolipid synthesis is coordinated with nutrient availability and cell growth in yeast cells, wherein TOR2 influences LCBs and ceramide synthesis. Ypk1/2 kinases act downstream of TOR2 to activate ceramide synthase activity. We observed that the deletions of these kinases in the background Cgyor1Δ resulted in enhanced azole susceptibility, confirming the involvement of these signaling pathways.

Molecular chaperone Hsp90 interacts with several client proteins to stabilize them or activate them, and through its interactions it generates a profound effect on the cellular stress response (67). The Hsp90 molecule, which is regulated by CK2 kinases, regulates Hog1 by phosphorylation. In the Cg*Yor1*Δ strain, the reduced phosphorylation of CgHog1 is indicative of suppressed CgHsp90 activity. Hsp90 also regulates resistance to both azoles and echinocandins by stabilizing the protein phosphatase calcineurin in C. albicans (87, 88). Calcineurin, which is a conserved Ca^2+^-calmodulin-activated protein phosphatase, has several functions related to calcium-dependent signaling and regulates several important cellular processes in yeasts. The role of the calcineurin pathway in azole resistance and virulence is well established in C. albicans and Candida dubliniensis (89). The calcineurin and *crz1* mutants in these yeasts show reduced tolerance to azoles and echinocandins (90, 91). We also observed that the Cg*yor1*Δ mutant is susceptible to calcineurin inhibitors. The deletion and phenotypes of CK2 regulatory subunits in the Cg*cdr1*Δ/Cg*yor1*Δ background reverted its azole-susceptible phenotype, which hints at suppressed calcineurin in the mutant.

**FIG 7:**
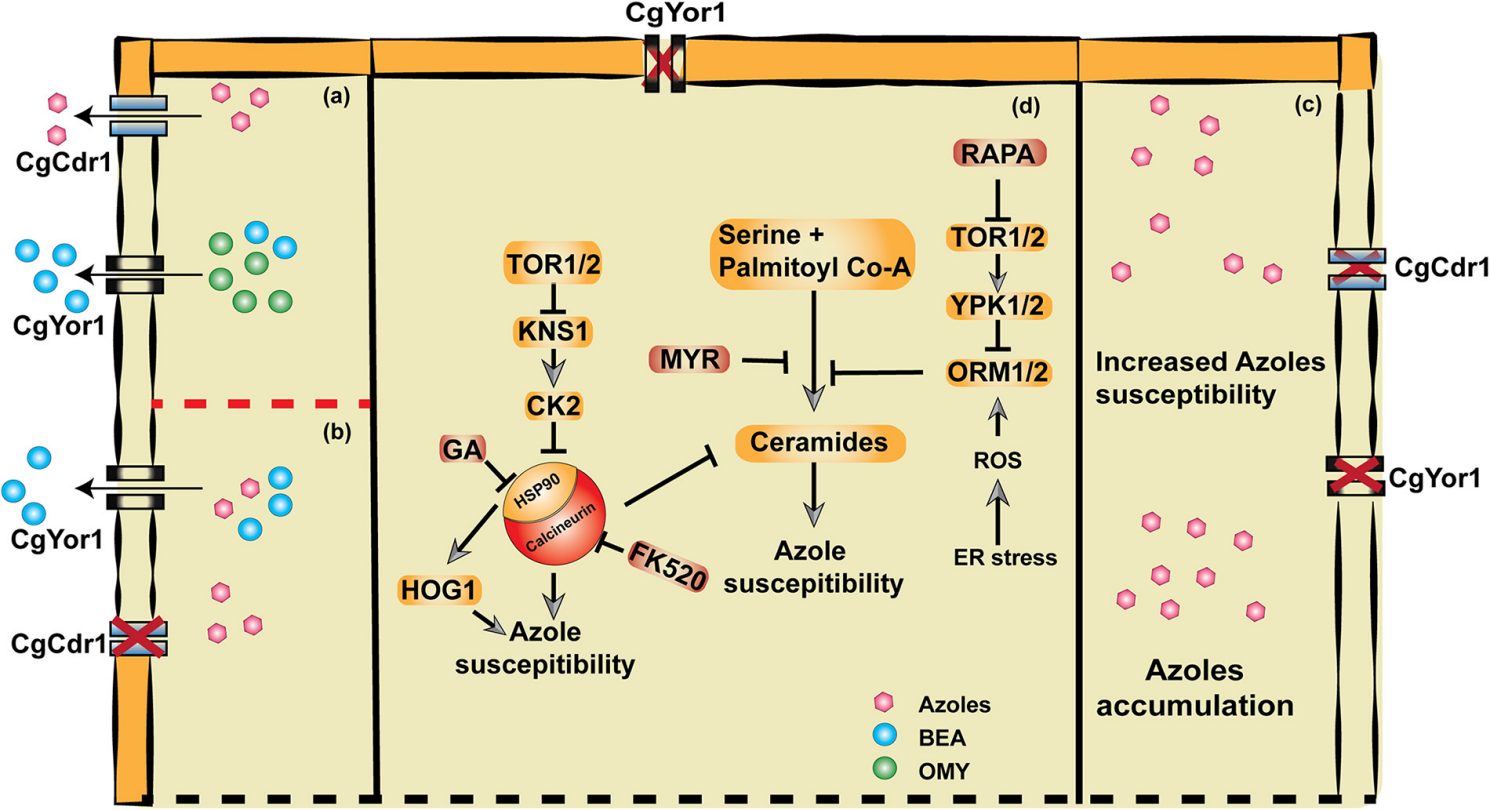
Model to explain the functional characterization of CgYor1. (a) Under normal growth conditions, when WT cells of C. glabrata encounter antifungals, CgCdr1 extrudes azoles and CgYor1 extrudes BEA. (b) In the absence of CgCdr1, the cells become susceptible to azoles. (c) In the absence of both the transporters CgCdr1 and CgYor1, enhanced susceptibility with azoles was observed, which could be due to increased accumulation of azoles in the strain. (d) Schematic diagram explaining azole susceptibility in Cg*yor1*Δ involving TOR and calcineurin cascades.

In conclusion, we are tempted to speculate that the deletion of Cg*YOR*1 results in perturbation of the membrane lipid composition that changes diffusion of FLC, leading to enhanced accumulation of drug and increased susceptibility to azoles. Noticeably, we find that the contribution of CgYor1 to azole susceptibility is independent of the major transporter CgCdr1. However, when CgCdr1 is present, it masks the effects of CgYor1. Importantly, CgYor1 does not transport OMY and FLC but contributes to azole resistance via ROS generation, suppressed TOR, and calcineurin signaling linked to altered sphingolipid homeostasis (Fig. 7). The accurate measurement of membrane composition and organization in CgYor1 cells and how selective CgYor1 deletion triggers the stress pathway are some aspects that remain to be evaluated. Nonetheless, the work highlights that, unlike other homologues, CgYor1 does not function as an OMY exporter in C. glabrata cells but contributes exclusively to azole resistance by activating stress signaling pathways.

## MATERIALS AND METHODS

### Chemicals and strains.

Antifungal drugs were of analytical grade and were purchased from Sigma. Yeast strains were maintained in yeast extract peptone dextrose (YPD) medium and in synthetically complete (SC) medium (0.67% yeast nitrogen base, 2% glucose and amino acids), wherever needed. Bacterial cultures were maintained in Luria-Bertani (LB) medium with the final concentration of ampicillin at 100 μg/mL, if needed. Bacterial strain Escherichia coli DH5α was used to maintain plasmids. Yeast extract peptone glycerol (YPG) medium with 3% glycerol was used to confirm petite colonies.

### Transporter deletions, revertant strain, and complementation strain construction.

The wild-type (WT) strain was C. glabrata BG14, which lacked the *ura3* gene. Sequentially multiple deletions were done by using a drug-based recyclable (FRT-NAT1-FRT) cassette, followed by the selection of mutants on the selection plate containing nourseothricin. A homologous recombination-based strategy was used to make deletions as described previously (27). The constructed strains and their respective genotypes are listed in Table S1.

The revertant strain was constructed by the episomal expression of the deleted genes. The modified plasmid pGRB2.3_HphB was made by replacing the *URA3* gene with the *HphB* gene and was then used as the parent plasmid for constructing revertant plasmids. The ABC transporter genes were cloned in plasmid pGRB2.3_HphB under their own promoter by using Gibson assembly. The deletion strains were then transformed with their respective plasmid and confirmed by PCR. Cg*YOR1* complementation in Sc*yor1*Δ was performed by expressing Cg*YOR1* in the expression plasmid pGPD2 under the GAPDH promoter. The plasmid constructs are listed in Table S2.

10.1128/mbio.03545-21.6 TABLE S2 Plasmids used in the study. Download Table S2, DOCX file, 0.01 MB. Copyright © 2022 Kumari et al. 2022 Kumari et al. https://creativecommons.org/licenses/by/4.0/ This content is distributed under the terms of the Creative Commons Attribution 4.0 International license.

### Drug susceptibility, growth curve, and synergistic assays.

The broth microdilution method for detection of MIC, spot dilution assays, and growth kinetics assessments were employed to examine drug susceptibility of these mutants. For this, different classes of antifungals, viz., azoles (fluconazole [FLC], ketoconazole [KTC], voriconazole [VRC], and miconazole [MCZ]), echinocandin (caspofungin [CSF]), polyene (amphotericin B [AmB]), allylamine (terbinafine [TER]), protein synthesis inhibitors (cycloheximide [CHX] and anisomycin [ANY]), DNA damaging agents (4-nitroquinoline, 4-NQO), and metal chelator (1,10-phenanthrolin [OP]) were used.

Susceptibility and synergy to numerous drugs were estimated either by broth microdilution or serial dilution spot assay as described previously (92). The MIC was determined after growing the cells at 30°C in YPD liquid medium for 48 h and taking readings at a wavelength of 600 nm by spectrometer. The MIC_80_ was defined at the lowest concentration inhibiting growth by at least 80% relative to the drug-free YPD control after incubation. For the spot assay, cell suspensions were 10-fold serially diluted in 0.9% saline, and a 3-μL aliquot was spotted on YPD with and without the drug. Agar plates were incubated at 30°C, and growth was recorded after 24 h. All MIC tests and spot tests were undertaken in triplicate. The growth curve assay was undertaken with YPD and using the microcultivation method at 30°C in 96-well plates as described previously (27). The fractional inhibitory concentration index (FICI) was calculated using the following formula: FICI = (MICAcombi/MICAalone) + (MICBcombi/MICBalone). A FICI of ≤0.5 was defined as synergy, a FICI of >0.5 but ≤4.0 was defined as no interaction, and a FICI of >4.0 was defined as antagonism.

For susceptibility profiling under N-limiting conditions, cells were initially grown in complete medium and then transferred into yeast nitrogen base (YNB) medium without amino acid and ammonium sulfate for 12 h. Serial dilutions of the cells were made and spotted on SC complete medium.

### R6G efflux and accumulation assay and substrate competition assay.

Spectrofluorometric-based R6G efflux was observed as described previously (27). Briefly, yeast strains were cultivated in YPD liquid medium at 200 rpm (30°C) for 16 h, and then secondary culture was grown until mid-log phase. The cells were harvested and washed twice with phosphate-buffered saline (PBS). Cells were suspended in PBS and incubated at 200 rpm (30°C) for 3 h under starvation (glucose free) conditions to reduce ABC pump activity. Cells were then washed twice and diluted to 10^8^ cells/mL in PBS. R6G was added at a concentration of 10 μM, and cells were incubated for a further 2 h at 200 rpm (30°C); for the accumulation assay, the supernatants were taken and analyzed with a FACSCalibur flow cytometer (FITC-A filter) using FlowJo v10.3 software. For the efflux assay, 2% glucose was provided as an energy source and incubated for 45 min. After incubation, the supernatants were analyzed with a spectrofluorometer at 527 nm excitation and 555 nm emission. For the substrate competition experiment, the tested substrates BEA, OMY, MYR, GA, FK520, and RAPA, when necessary, were added at a concentration of about 10× MIC along with R6G.

### ^3^H-FLC accumulation assay.

The ^3^H-FLC accumulation assay was performed as described previously (93). Overnight-grown (16- h grown culture) samples were washed three times with YNB, starved of glucose for 3 h, and then treated with ^3^H-FLC in YNB ± 2% glucose in technical triplicate. Samples were incubated with shaking at room temperature for 24 h. At 24 h, the optical density at 600 nm (OD_600_) of each sample was taken, and then a stop solution, consisting of YNB + FLC (20 μM), was mixed with each sample. Then samples were poured over glass microfiber filters on a vacuum and washed again with YNB. Filters with washed cells were placed in scintillation fluid and counted. Values were adjusted to CPM per 10^8^ cells based on the OD_600_ of each sample recorded right before filtering. For ^3^H-FLC efflux measurements, after 24 h, ^3^H-FLC preloading samples were washed and resuspended in fresh YNB ± 2% glucose, and then intracellular ^3^H-FLC was measured at the designated time intervals.

### Measurement of ROS levels.

The oxidant-sensitive probe DCFDA was used to measure endogenous ROS. Cells were set to an OD_600_ of 0.1 in YPD medium and incubated for 4 h at 30°C with shaking at 200 revolutions (rev)/min. Then, DCFDA was added at a final concentration of 2 μM, and the culture was grown for 2 h. The cells were then analyzed with a FACSCalibur flow cytometer at 495 nm excitation and 529 nm emission (FITC-A filter) and analyzed with FlowJo v10.3 software.

### Ergosterol measurement and sphingolipidome.

Ergosterol was measured by using a UV-spectrometer as described previously (94). Lipid extraction was performed with the Mandala formulation followed by Bligh and Dyer’s extraction and base hydrolysis as described earlier (95). Sphingolipids were analyzed by using an LC-MS/MS assay using the QTRAP 4500 (SCIEX, USA) as previously described (82). Data were normalized by ng of sphingolipids/mg of total protein and converted into mole%.

### Total RNA isolation and cDNA synthesis and quantitative real-time PCR.

Total RNA was extracted by using the RNeasy minikit (Qiagen, Germany). Briefly, overnight-grown cultures of C. glabrata were diluted to 0.1 OD_600_ and grown for 4 h at 30°C in YPD. Cells were then washed with PBS, and total RNA was extracted as per the manufacturer’s protocol. RNA samples were quantified using a nanodrop 2000 spectrophotometer (Thermo Scientific, USA), and 5 μg of total RNA was used for cDNA synthesis using a RevertAid H minus first-strand cDNA synthesis kit (Thermo Scientific, Lithuania).

The quantitative gene expression profile was evaluated by using a DyNAmo Flash SYBR green qPCR kit (Thermo Scientific, Lithuania) and gene-specific primers, including CgPGK1, for normalization in the CFX96TM real-time PCR system (Bio-Rad, USA). The expression level of the tested genes was measured by comparing the threshold cycle (*C_T_*) value of the gene with CgPGK1. Comparative expression profiles were analyzed with the 2^-ΔΔ^*^CT^* method. qRT-PCR was performed in biological duplicate with technical triplicate.

### Western blot analysis.

Western blotting of CgHog1 was performed as described previously (22). Briefly, log-phase-grown C. glabrata cells were pelleted down at 5,000 rpm for 10 min and immediately frozen in liquid nitrogen. Cells were lysed using the lysis buffer (50 mM Tris-Cl [pH 7.5]; 1% sodium deoxycholate; 1% Triton X-100; 0.1% SDS; 50 mM sodium fluoride; 0.1 mM sodium vanadate; 5 mM sodium pyrophosphate; 0.05% phenylmethylsulfonyl fluoride [PMSF], cOmplete EDTA free protease inhibitor cocktail [Roche] 1 tablet per 10 mL). The pelleted cells were lysed in 200 μL of lysis buffer with glass beads by 10 vigorous vortex cycles (1 min vortex with 1 min incubation in ice), and the resulting homogenate was centrifuged at 13,500 × *g* for 10 min, and the protein concentration of the supernatant was estimated using Bradford’s method. Then, 20 μg protein sample was loaded and resolved by 10% SDS polyacrylamide gel electrophoresis and then blotted onto a nitrocellulose membrane. Phosphorylated CgHog1 protein was detected using anti-dually phosphorylated p38 (α-phospho Hog1) antibody (Cell Signaling Technology), and total CgHog1 protein was detected with anti-Hog1 antibody (Y-215; Santa Cruz Biotechnology). Band intensities were quantified through Image Lab software (Bio-Rad); 0.5 M NaCl was used as osmotic stress to visualize the phosphorylation level of CgHog1. Total CgHog1 was used as a loading control.

### Statistical analysis.

All analyses were carried out in triplicate forms. Calibration curves were determined using Microsoft Excel. Student’s *t* test was used for statistical significance analysis. Differences of *P < *0.05 were considered significant.
