# Supplementary figures and images for: Unmasking of CgYor1-Dependent Azole Resistance Mediated by Target of Rapamycin (TOR) and Calcineurin Signaling in Candida glabrata
Source: mBio. 2022 Jan 18;13(1):e03545-21. doi: 10.1128/mbio.03545-21 (PMC8764518; doi:10.1128/mbio.03545-21)

## Slide 1
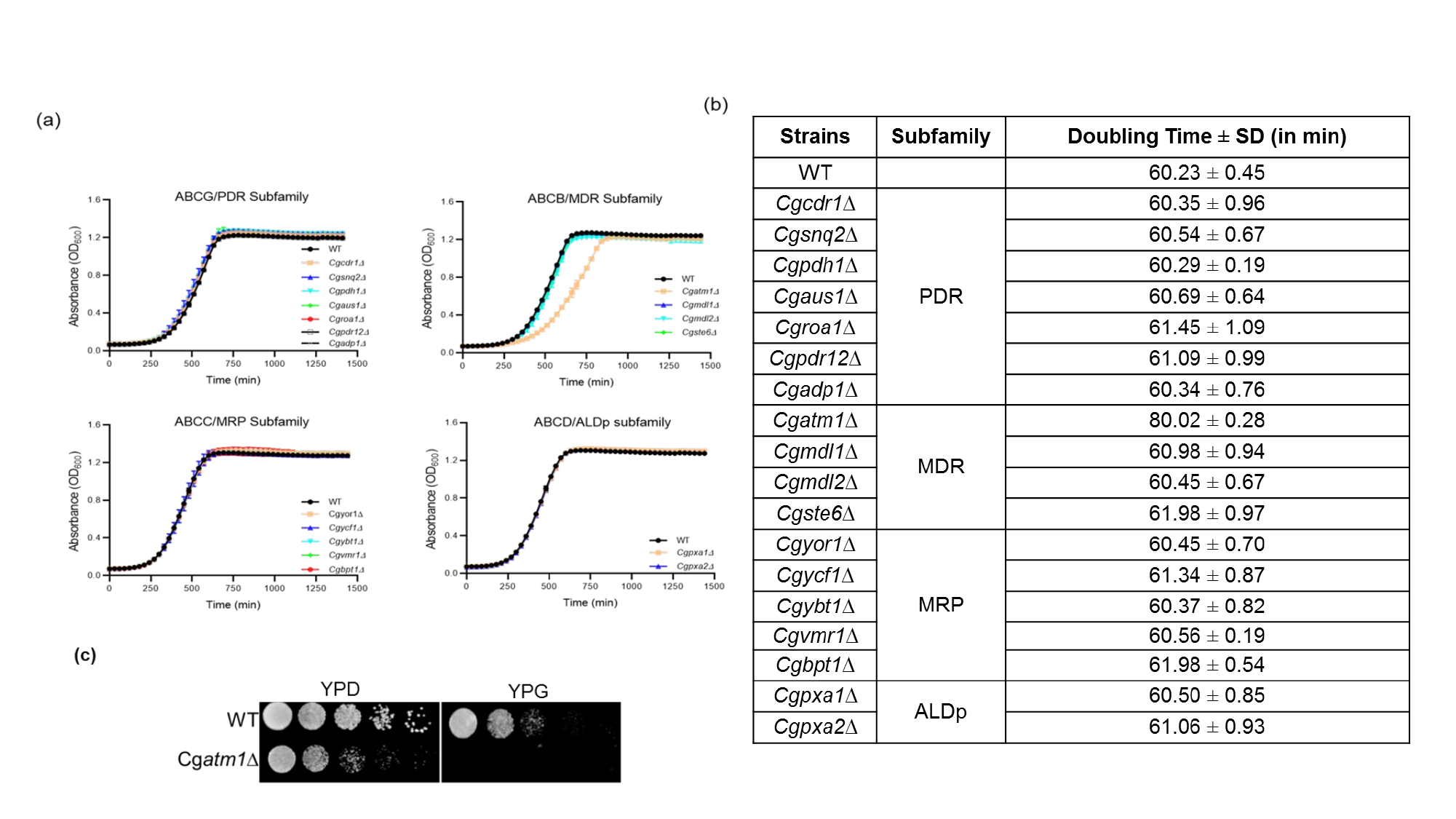

Supplement: FIG S1 [file mbio.03545-21-sf001.pptx]

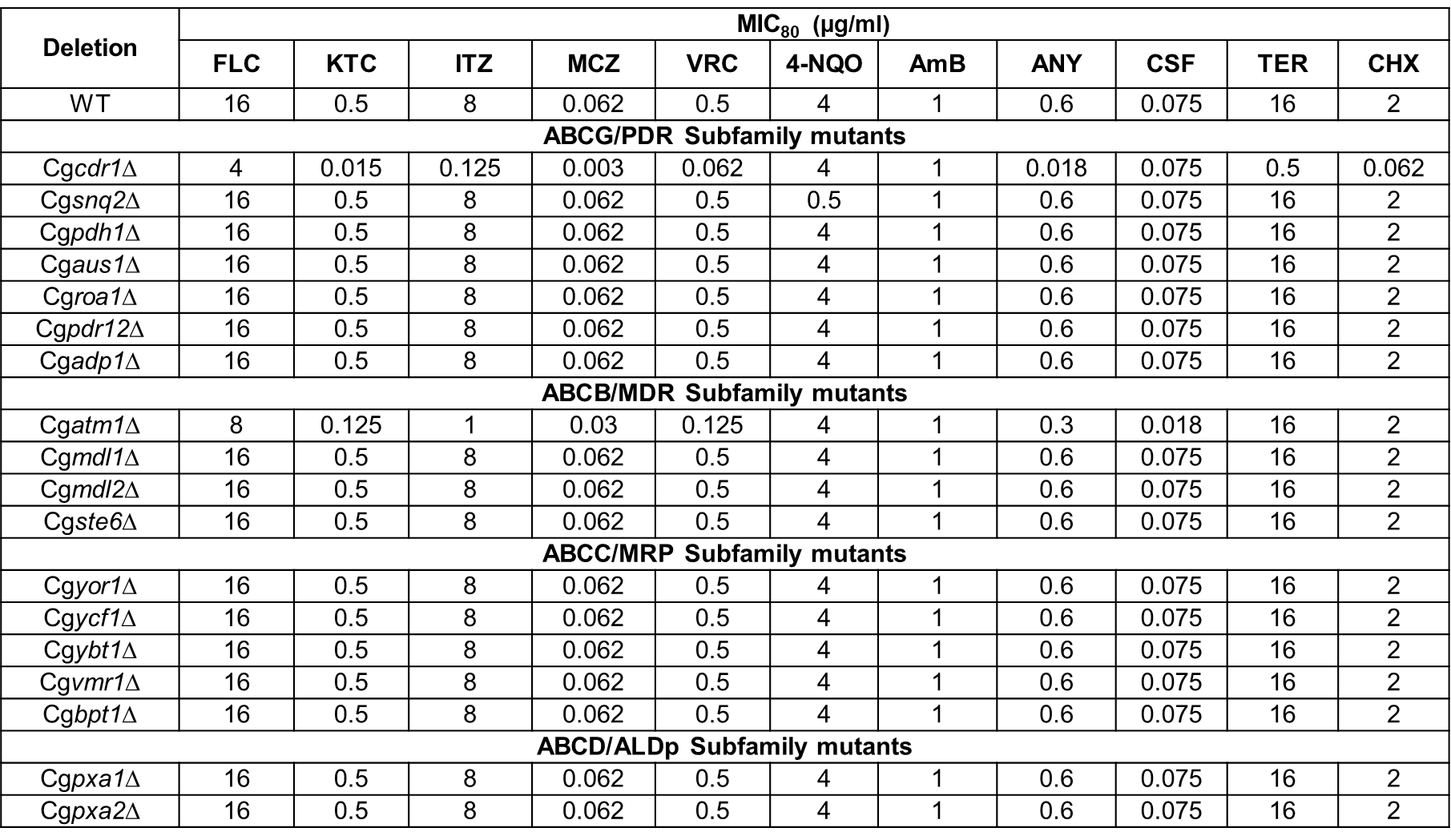

Supplement: TABLE S3 [file mbio.03545-21-st003.docx]

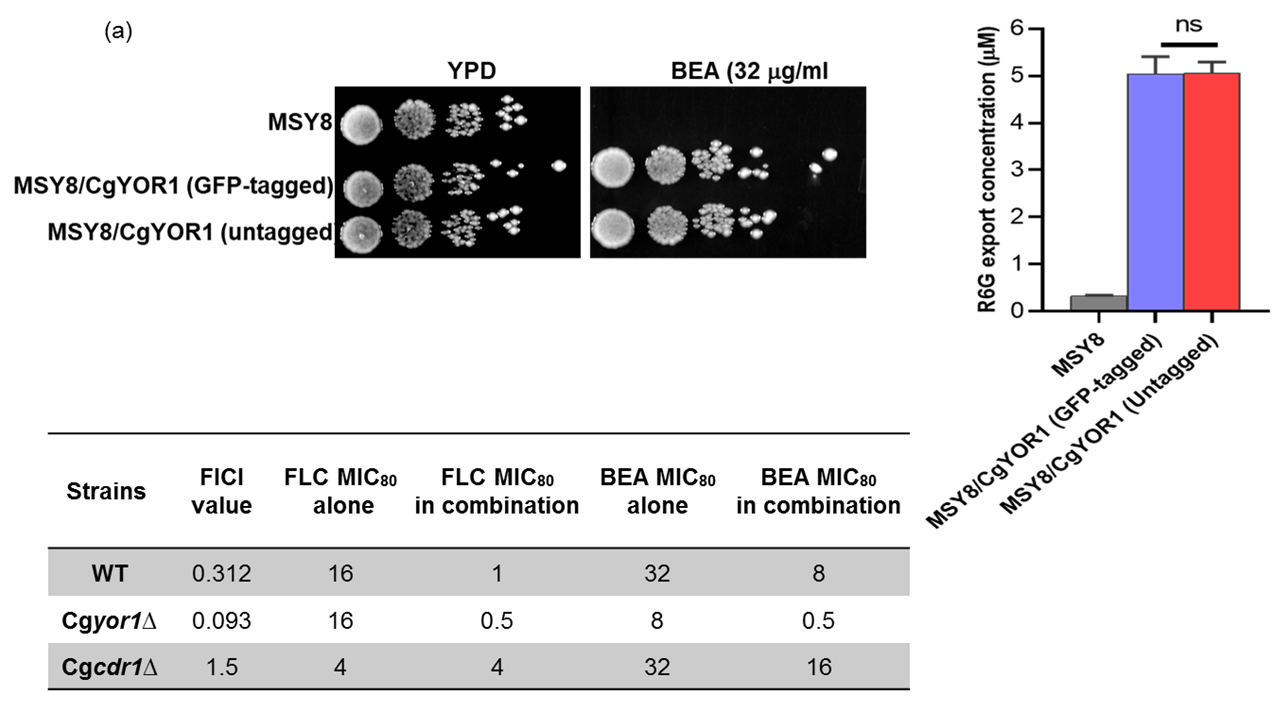

Supplement: FIG S2 [file mbio.03545-21-sf002.tif]

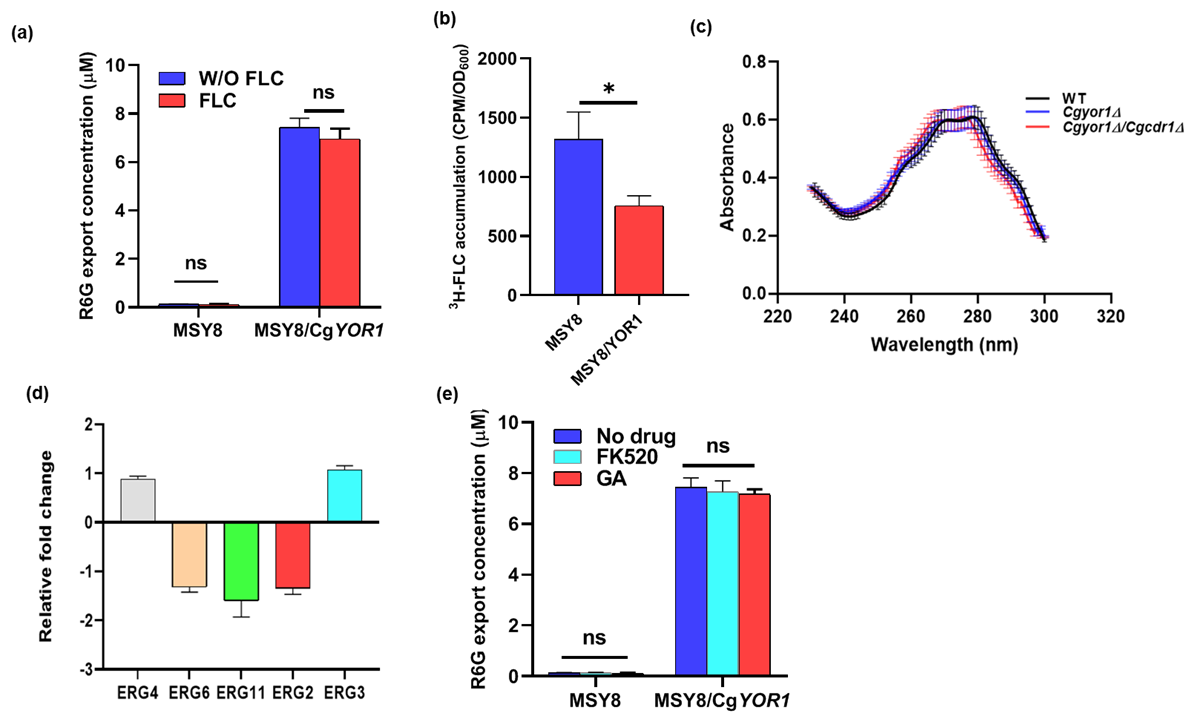

Supplement: FIG S3 [file mbio.03545-21-sf003.tif]

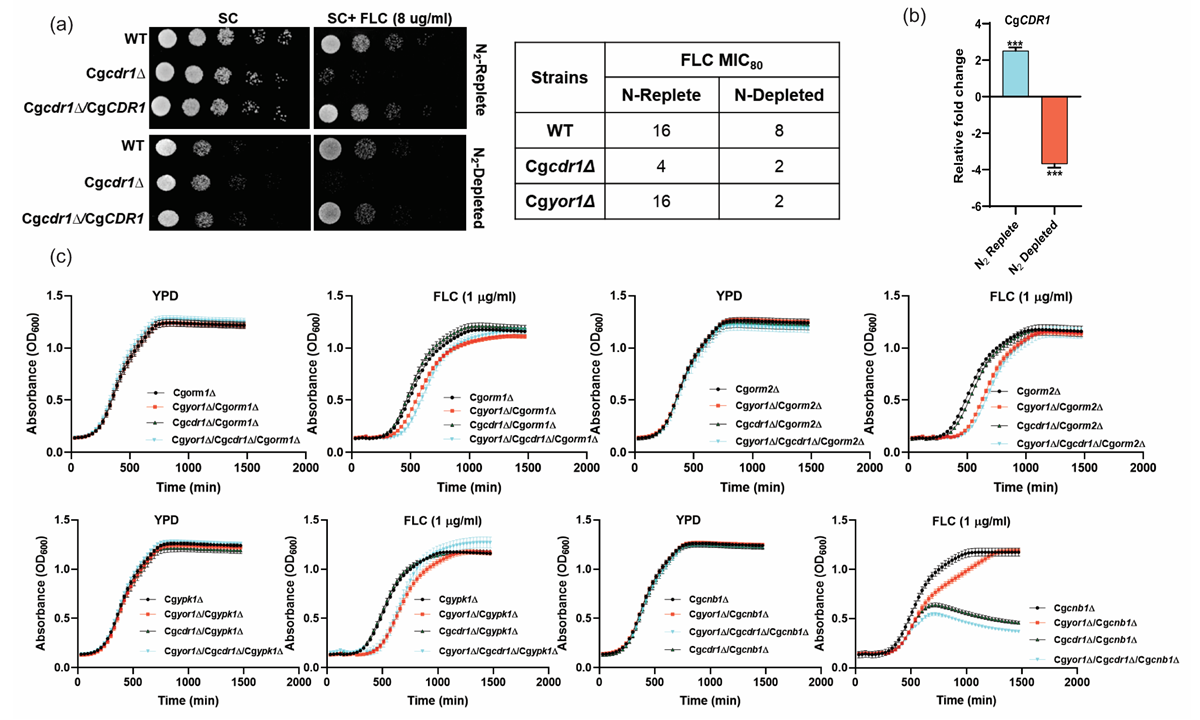

Supplement: FIG S4 [file mbio.03545-21-sf004.tif]
