# Supplementary material for: Unmasking of CgYor1-Dependent Azole Resistance Mediated by Target of Rapamycin (TOR) and Calcineurin Signaling in Candida glabrata
Source: mBio. 2022 Jan 18;13(1):e03545-21. doi: 10.1128/mbio.03545-21 (PMC8764518; doi:10.1128/mbio.03545-21)
Supplement: TABLE S1 [file mbio.03545-21-st001.docx]

| **Strains** | **Genotype** | **Source/Reference** |
| --- | --- | --- |
| WT | BG14 | Lab Stock |
| MSY8 | *∆Cgsnq2::FRT/∆Cgaus1::FRT/∆Cgcdr1::FRT/ ∆Cgpdh1::FRT/∆Cgycf1::FRT/∆Cgybt1::FRT/ ∆Cgyor1::FRT/Cgpdr1::CgPDR1^G840C^ FRT* | Kumari et al., 2020 |
| BY4741 | MATa his3Δ1 leu2Δ0 met15Δ0 ura3Δ0 | Lab stock |
| Scyor1∆ | *BY4741/Scyor1∆::KanMX* | Lab stock |
| *Cgsnq2*∆ | *∆Cgsnq2::NAT1* | This study |
| *Cgaus1∆* | *∆Cgaus1::NAT1* | This study |
| *Cgcdr1∆* | *∆Cgcdr1::NAT1* | This study |
| *Cgpdh1∆* | *∆Cgpdh1::NAT1* | This study |
| *Cgroa1∆* | *∆Cgroa1::NAT1* | This study |
| *Cgpdr12∆* | *∆Cgpdr12::NAT1* | This study |
| *Cgadp1∆* | *∆Cgadp1::NAT1* | This study |
| *Cgste6∆* | *∆Cgste6::NAT1* | This study |
| *Cgmdl1∆* | *∆Cgmdl1::NAT1* | This study |
| *Cgmdl2∆* | *∆Cgmdl2::NAT1* | This study |
| *Cgatm1∆* | *∆Cgatm1::NAT1* | This study |
| *Cgyor1∆* | *∆Cgyor1::NAT1* | This study |
| *Cgycf1∆* | *∆Cgycf1::NAT1* | This study |
| *Cgybt1∆* | *∆Cgybt1::NAT1* | This study |
| *Cgvmr1∆* | *∆Cgvmr1::NAT1* | This study |
| *Cgbpt1∆* | *∆Cgbpt1::NAT1* | This study |
| *Cgpxa1∆* | *∆Cgpxa1::NAT1* | This study |
| *Cgpxa2∆* | *∆Cgpxa2::NAT1* | This study |
| *Cgcdr1∆*/*Cgaus1∆* | *∆Cgcdr1::FRT/∆Cgaus1::NAT1* | This study |
| *Cgcdr1∆*/*Cgsnq2∆* | *∆Cgcdr1::FRT/∆Cgsnq2::NAT1* | This study |
| *Cgcdr1∆*/*Cgybt1∆* | *∆Cgcdr1::FRT/∆Cgybt1::NAT1* | This study |
| *Cgcdr1∆*/*/Cgycf1∆* | *∆Cgcdr1::FRT/∆Cgycf1::NAT1* | This study |
| *Cgcdr1∆*/*Cgpdh1∆* | *∆Cgcdr1::FRT/∆Cgpdh1::NAT1* | This study |
| *Cgcdr1∆*/*Cgyor1∆* | *∆Cgcdr1::FRT/∆Cgyor1::NAT1* | This study |
| *Cgyor1∆*/*Cgpdh1∆* | *∆Cgyor1::FRT/∆Cgpdh1::NAT1* | This study |
| *Cgyor1∆*/*Cgsnq2∆* | *∆Cgyor1::FRT/∆Cgsnq2::NAT1* | This study |
| *Cgyor1∆*/*Cgaus1∆* | *∆Cgyor1::FRT/∆Cgaus1::NAT1* | This study |
| *Cgyor1∆*/*Cgycf1∆* | *∆Cgyor1::FRT/∆Cgycf1::NAT1* | This study |
| *Cgyor1∆*/*Cgybt1∆* | *∆Cgyor1::FRT/∆Cgybt1::NAT1* | This study |
| *Cgckb1∆* | *∆Cgckb1::NAT1* | This study |
| *Cgckb2∆* | *∆Cgckb2::NAT1* | This study |
| *Cgcdr1∆*/*Cgckb1∆* | *∆Cgcdr1::FRT/ ∆Cgckb1::NAT1* | This study |
| *Cgcdr1∆*/*Cgckb2∆* | *∆Cgcdr1::FRT/ ∆Cgckb2::NAT1* | This study |
| *Cgyor1∆*/*Cgckb1∆* | *∆Cgyor1::FRT/ ∆Cgckb1::NAT1* | This study |
| *Cgyor1∆*/*Cgckb2∆* | *∆Cgyor1::FRT/ ∆Cgckb2::NAT1* | This study |
| *Cgcdr1∆*/*Cgyor1∆*/*Cgckb1∆* | *∆Cgcdr1::FRT/∆Cgyor1::FRT/ ∆Cgckb1::NAT1* | This study |
| *Cgcdr1∆*/*Cgyor1∆*/*Cgckb2∆* | *∆Cgcdr1::FRT/∆Cgyor1::FRT/ ∆Cgckb1::NAT1* | This study |
| *Cgyor1∆*/*Cgcnb1∆* | *∆Cgyor1::FRT/ ∆Cgcnb1::NAT1* | This study |
| *Cgyor1∆*/*Cgypk1∆* | *∆Cgyor1::FRT/ ∆Cgypk1::NAT1* | This study |
| *Cgyor1∆*/*Cgypk2∆* | *∆Cgyor1::FRT/ ∆Cgypk2::NAT1* | This study |
| *Cgcdr1∆*/*Cgcnb1∆* | *∆Cgcdr1::FRT/ ∆Cgcnb1::NAT1* | This study |
| *Cgcdr1∆*/*Cgypk1∆* | *∆Cgcdr1::FRT/ ∆Cgypk1::NAT1* | This study |
| *Cgcdr1∆*/*Cgypk2∆* | *∆Cgcdr1::FRT/ ∆Cgypk2::NAT1* | This study |
| *Cgcnb1∆* | *∆Cgcnb1::NAT1* | This study |
| *Cgypk1∆* | *∆Cgypk1::NAT1* | This study |
| *Cgypk2∆* | *∆Cgypk2::NAT1* | This study |
| Scyor1∆/CgYOR1 | *BY4741/Scyor1∆::KanMX/pGPD2_CgYOR1* | This study |
| Cgyor1∆/CgYOR1 | *∆Cgyor1::FRT/ pGRB2.3_HphB_CgYOR1* | This study |
| Cgcdr1∆/CgCDR1 | *∆Cgcdr1::FRT/ pGRB2.3_HphB_CgCDR1* | This study |
