# Supplementary material for: Unmasking of CgYor1-Dependent Azole Resistance Mediated by Target of Rapamycin (TOR) and Calcineurin Signaling in Candida glabrata
Source: mBio. 2022 Jan 18;13(1):e03545-21. doi: 10.1128/mbio.03545-21 (PMC8764518; doi:10.1128/mbio.03545-21)
Supplement: TABLE S4 [file mbio.03545-21-st004.docx]

| **Sphingolipid species** | **WT** | | **Cg*yor1*∆** | | **Cgcdr*1*∆** | | **Cgcdr1∆/Cg*yor1*∆** | |
| --- | --- | --- | --- | --- | --- | --- | --- | --- |
|  | **Average** | **SD** | **Average** | **SD** | **Average** | **SD** | **Average** | **SD** |
| DHS | 0.646 | 0.190 | 0.631 | 0.620 | 0.526 | 0.068 | 0.522 | 0.145 |
| DHS1P | 0.013 | 0.023 | 0.035 | 0.027 | 0.002 | 0.004 | 0.033 | 0.031 |
| SPH | 0.055 | 0.015 | 0.093 | 0.079 | 0.059 | 0.038 | 0.098 | 0.051 |
| SPH1P | 0.004 | 0.008 | 0.005 | 0.005 | 0.006 | 0.005 | **0.002** | **0.001** |
| PHS | 0.601 | 0.158 | 0.432 | 0.338 | 0.551 | 0.153 | 0.414 | 0.061 |
| PHS1P | 0.016 | 0.024 | 0.000 | 0.000 | 0.000 | 0.000 | 0.002 | 0.002 |
| Cer(d18:0/18:0) | 0.095 | 0.028 | 0.073 | 0.047 | 0.086 | 0.050 | **0.124** | **0.027** |
| Cer(d18:0/24:0) | 0.507 | 0.195 | 0.316 | 0.067 | 0.344 | 0.179 | 0.547 | 0.279 |
| Cer(d18:0/26:0) | 0.451 | 0.187 | 0.236 | 0.115 | 0.336 | 0.044 | 0.488 | 0.182 |
| Cer(d18:0/28:0) | 0.281 | 0.083 | 0.214 | 0.050 | 0.327 | 0.076 | **0.645** | **0.034** |
| Cer(d18:1/18:0) | 0.003 | 0.005 | 0.003 | 0.006 | 0.000 | 0.000 | 0.005 | 0.008 |
| Cer(d18:1/24:0) | 0.273 | 0.103 | **0.117** | **0.021** | 0.243 | 0.180 | 0.312 | 0.136 |
| Cer(d18:1/26:0) | 0.120 | 0.106 | 0.018 | 0.046 | 0.164 | 0.172 | 0.173 | 0.127 |
| Cer(d18:1/28:0) | 0.073 | 0.059 | 0.044 | 0.001 | **0.094** | **0.035** | 0.127 | 0.068 |
| Cer(d18:1/18:0(2OH)) | 0.206 | 0.223 | 0.060 | 0.061 | **0.198** | **0.067** | 0.343 | 0.298 |
| Cer(d18:1/24:0(2OH)) | 0.750 | 0.428 | 0.272 | 0.273 | 0.489 | 0.404 | 0.852 | 0.411 |
| Cer(d18:1/26:0(2OH)) | 0.412 | 0.183 | 0.270 | 0.224 | 0.395 | 0.135 | **0.854** | **0.391** |
| Cer(t18:0/18:0) | 0.042 | 0.006 | **0.012** | **0.008** | 0.023 | 0.014 | 0.033 | 0.012 |
| Cer(t18:0/24:0) | 9.508 | 1.090 | 9.371 | 2.225 | 9.707 | 2.934 | 9.237 | 0.615 |
| Cer(t18:0/26:0) | 41.209 | 3.571 | **37.061** | **1.146** | 38.590 | 1.577 | 37.105 | 1.229 |
| Cer(t18:0/28:0) | 33.302 | 1.863 | **40.420** | **2.161** | 35.261 | 3.236 | 37.209 | 1.446 |
| Cer(t18:0/18:0(2OH)) | 0.023 | 0.040 | 0.044 | 0.031 | 0.029 | 0.010 | **0.048** | **0.013** |
| Cer(t18:0/24:0(2OH)) | 1.137 | 0.330 | 0.834 | 0.320 | 1.013 | 0.391 | 0.928 | 0.179 |
| Cer(t18:0/26:0(2OH)) | 9.491 | 0.773 | 9.059 | 1.410 | **11.075** | **0.544** | 8.839 | 0.503 |
| Cer(t18:0/28:0(2OH)) | 0.158 | 0.046 | 0.184 | 0.081 | 0.214 | 0.071 | 0.144 | 0.008 |
