# Supplementary material for: Unmasking of CgYor1-Dependent Azole Resistance Mediated by Target of Rapamycin (TOR) and Calcineurin Signaling in Candida glabrata
Source: mBio. 2022 Jan 18;13(1):e03545-21. doi: 10.1128/mbio.03545-21 (PMC8764518; doi:10.1128/mbio.03545-21)
Supplement: Supplementary file 4 [file mbio.03545-21-st002.docx]

Plasmids	Source
pGRB2.3	Addgene#45343
pGPD2	Addgene#43972
pMS6	Kumari et al., 2020
pGRB2.3_HphB	This study
pGRB2.3_HphB/CgYOR1	This study
pGRB2.3_HphB/CgCDR1	This study
pMS6/CgYOR1	This study
pMS6/CaYOR1	This study
pMS6/ScYOR1	This study
pGPD2/CgYOR1	This study
